# Time-Course of Physiological Adaptations to High-Intensity Interval Training-Based Cardiac Rehabilitation After Myocardial Infarction

**DOI:** 10.3390/jcm15124545

**Published:** 2026-06-11

**Authors:** Kristina Skroce, Dijana Travica Samsa, Marina Njegovan, Damjan Dusevic, Andrej Belancic, Cantor Tarperi, Federico Schena, Viktor Persic

**Affiliations:** 1 Faculty of Medicine, University of Rijeka, 51000 Rijeka, Croatiaviktor.persic@medri.uniri.hr (V.P.); 2 Hospital for Medical Rehabilitation of Heart and Lung Diseases and Rheumatism Thalassotherapia-Opatija, 51410 Opatija, Croatiadamjandadodusevic@gmail.com (D.D.); 3 Faculty of Health Studies, University of Rijeka, 51000 Rijeka, Croatia; 4 Department of Basic and Clinical Pharmacology and Toxicology, Faculty of Medicine, University of Rijeka, 51000 Rijeka, Croatia; a.belancic93@gmail.com; 5 Department of Neurosciences, Biomedicine and Movement Sciences, University of Verona, 37131 Verona, Italy; cantor.tarperi@univr.it (C.T.); federico.schena@univr.it (F.S.); 6 Faculty of Dental Medicine and Health Osijek, Josip Juraj Strossmayer University of Osijek, 31000 Osijek, Croatia

**Keywords:** cardiac rehabilitation, high-intensity interval training, myocardial infarction, NT-proBNP, exercise

## Abstract

**Background**: High-intensity interval training (HIIT) is increasingly used in exercise-based cardiac rehabilitation (ebCR) after myocardial infarction (MI), yet the temporal sequence of physiological, cardiac, biochemical, and functional adaptations remains incompletely characterized. **Methods**: Stable post-STEMI (ST-segment elevation myocardial infarction, MI-group) and previously inactive participants without known cardiovascular, metabolic or systemic disease (CTRL group) completed 12-week supervised outpatient HIIT (4 × 4 min intervals at 85–90% HRpeak (peak heart rate), ~80–90% of VO_2_peak, 3 sessions/week). Assessments were performed at baseline (T1), 4 (T2), 8 (T3), and 12 weeks (T4), including cardiopulmonary exercise testing (CPET), echocardiography, blood biomarkers, body composition, six-minute walk test (6MWT), and RAND-36. Longitudinal changes were analyzed using Friedman tests with Dunn post hoc comparisons; between-group differences used Mann–Whitney U tests with Holm correction. **Results**: VO_2_peak increased significantly in both groups (*p* < 0.001), increasing by ~22% from T1 to T4 in MI (median 20.1 to 24.5 mL·kg^−1^·min^−1^) and ~23% from T1 to T4 in CTRL (median 22.3 to 27.6 mL·kg^−1^·min^−1^). LVEF (left ventricular ejection fraction) improved early in MI, increasing from 52.5% (50.0–55.0) at T1 to 57.5% (55.2–58.7) at T2 and up to 60% (55.8–60.0) at T4 (all *p* < 0.001), while LV dimensions remained stable. NT-proBNP (N-terminal pro-B-type natriuretic peptide) showed no significant longitudinal change (*p* = 0.510), and CRP (C-reactive protein) decreased from 2.1 to 0.7 mg·L^−1^ (*p* = 0.008) in MI. Both groups improved body fat % and 6MWT distance (*p* < 0.001). **Conclusions**: In low-risk stable post-STEMI patients, longitudinal changes during supervised HIIT-based ebCR were consistent with improved VO_2_peak and LVEF, without clinically relevant increases in cardiac stress biomarkers. However, due to the observational design and absence of clinical comparator groups, these findings should be interpreted as descriptive and support further evaluation in larger randomized studies.

## 1. Introduction

Exercise-based cardiac rehabilitation (ebCR) is a cornerstone of secondary prevention following myocardial infarction (MI) and is widely recognized as one of the most cost-effective interventions for improving outcomes across a broad spectrum of cardiovascular disease (CVD) [1]. Participation in ebCR has been shown to reduce cardiovascular mortality, morbidity, and disability, while simultaneously improving health-related quality of life [2].

Within ebCR, high-intensity interval training (HIIT) has become an increasingly studied modality for secondary prevention after MI and has recently emerged as an alternative or adjunct to traditional moderate-intensity continuous training (MICT) [3,4]. Interest in HIIT is largely driven by its ability to induce meaningful improvements in cardiorespiratory fitness during relatively short training sessions, while remaining feasible under supervised conditions. Notably, HIIT can achieve physiological adaptations comparable to those observed with MICT despite a lower total exercise volume [5].

Although ebCR is known to improve cardiorespiratory fitness and clinical outcomes after MI, many studies evaluate outcomes primarily at program completion, typically after interventions lasting ≥6 or 12 weeks [6,7,8]. Consequently, while the overall magnitude of benefit from structured exercise training, particularly HIIT, is well established, the temporal pattern of adaptation across different physiological systems remains incompletely characterized, as most cardiac rehabilitation studies rely on pre–post comparisons rather than repeated intermediate assessments. Evidence from endurance-training time-course studies indicates that key determinants of aerobic performance adapt at different rates [9]. For example, early improvements in submaximal exercise tolerance and peripheral efficiency may precede later changes in maximal aerobic capacity or resting cardiac function [10].

While most training studies focus on longer exercise, Moholdt et al. [11] demonstrated that as little as 4 weeks of interval training can significantly increase VO_2_peak in cardiac patients. Similarly, studies examining cardiac structural and functional adaptations typically employ intervention durations ranging from 4 to 52 weeks and generally report favorable outcomes [12], yet intermediate measurements are rarely performed, limiting insight into the progression of these adaptations. At the biochemical level, exercise training may favorably influence cardiometabolic risk factors and systemic inflammation, while cardiac stress biomarkers such as NT-proBNP offer complementary information regarding myocardial load and efficiency [13,14]. Importantly, recovery after MI encompasses not only physiological improvements but also meaningful gains in health-related quality of life [15].

Taken together, existing evidence indicates that HIIT as part of ebCR can improve cardiorespiratory fitness in patients with MI and CAD, including studies using 4 × 4 min interval protocols and repeated CPET-based assessments. However, evidence regarding adverse effects remains insufficient for definitive conclusions, and the concurrent time course of adaptation across multiple physiological systems remains incompletely characterized. In particular, the temporal relationship between CPET-derived adaptations, echocardiographic changes, biochemical markers, body composition, functional capacity, and patient-reported outcomes during HIIT-based outpatient ebCR has not been fully described. Therefore, the present study aimed to characterize the 4-weekly multidomain time course of physiological, cardiac, biochemical, functional, and patient-reported adaptations during a 12-week supervised HIIT-based outpatient ebCR in clinically stable, low-risk post-MI patients. Outcomes were compared with previously inactive participants without known cardiovascular, metabolic or systemic disease using repeated assessments of CPET parameters, echocardiography, biochemical markers, anthropometry, functional capacity, and quality of life.

## 2. Materials and Methods

### 2.1. Population and Training Groups

**Figure 1:**
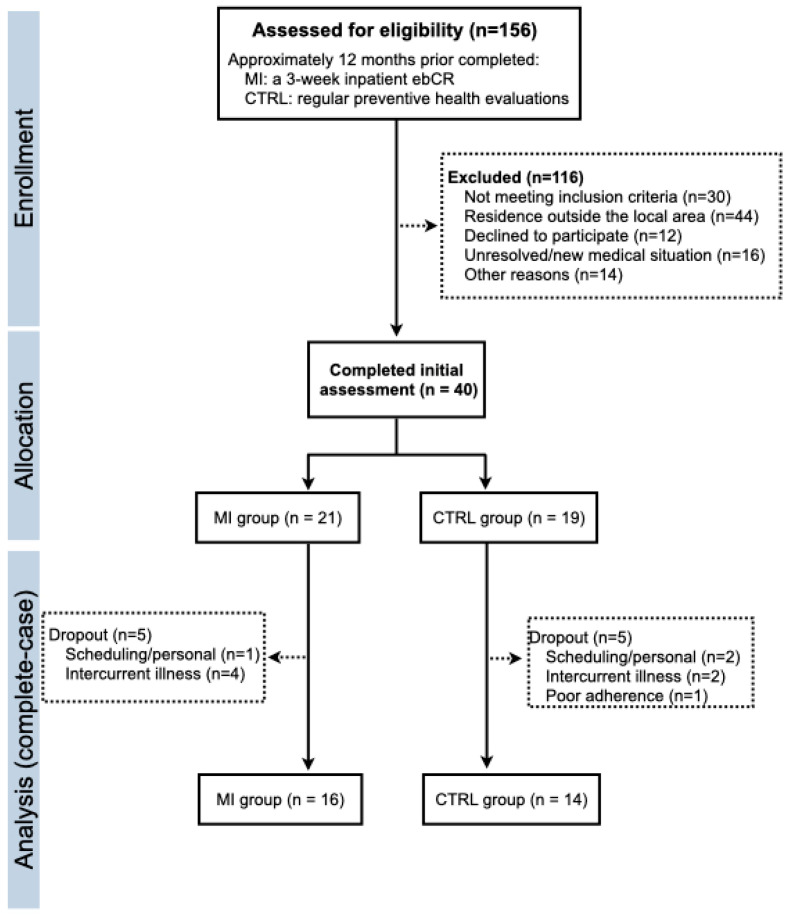
Flow of participants through the study. Participants were assessed for eligibility following completion of prior standard care. Post-STEMI patients had completed a standardized 3-week in-patient ebCR program approximately 12 months prior to enrollment, while control participants (CTRL) had undergone regular preventive health evaluations. After exclusions based on predefined criteria, eligible participants completed baseline assessments and were allocated to MI or CTRL groups. Both groups participated in a supervised 12-week HIIT-based exercise program. Participants who discontinued the intervention due to scheduling or personal reasons, intercurrent illness, or insufficient adherence were excluded from the longitudinal analysis.

Myocardial infarction group (MI) patients were eligible for inclusion if they had a confirmed diagnosis of ST-elevation myocardial infarction (STEMI), defined according to the 2023 ESC Guidelines as acute myocardial infarction with persistent ST-segment elevation on ECG and a rise and/or fall of high-sensitivity cardiac troponin with at least one value above the 99th percentile upper reference limit [16]. Eligible patients had a confirmed history of STEMI, had completed a standardized 3-week in-patient phase II ebCR program at Thalassotherapia-Opatija before enrollment, had remained physically inactive for at least 6 months prior to study inclusion, and were invited to participate in a subsequent supervised outpatient ebCR program. Individuals aged 18–80 years were included. The control group (CTRL) consisted of previously inactive participants without known cardiovascular, metabolic, or systemic disease comparable in age and sex. The control group was not designed as a non-intervention control, but rather as a reference model representing the physiological response to training under standard conditions. Importantly, the CTRL group was not intended to represent a clinical cardiac rehabilitation comparator. Participants were identified during routine preventive cardiovascular health evaluations, which included a comprehensive clinical examination and laboratory testing. Inclusion criteria for the control group required: absence of any known cardiovascular, metabolic, or systemic disease, no engagement in structured physical training or regular exercise for the past 6 months, aged between 18 and 80 years. Participants from both the MI and CTRL groups were excluded if they presented with uncontrolled arterial hypertension or if they exhibited any signs of clinically manifest heart failure or hemodynamic decompensation. Individuals with uncontrolled arrhythmias or persistent resting tachycardia (>100 beats per minute) were not eligible for participation. Likewise, subjects with uncontrolled metabolic disorders, including acute complications of diabetes mellitus, were excluded, as were those with significant comorbidities such as major endocrine, autoimmune, or malignant diseases, or a history of solid-organ transplantation. Additional exclusion criteria included the presence of clinically relevant peripheral arterial disease, current treatment with anticoagulant therapy, and age outside the predefined range of 18 to 80 years. Current anticoagulant therapy was an exclusion criterion as part of the conservative eligibility criteria used to define a clinically stable, low-risk post-STEMI cohort for this pilot protocol. Further exclusion factors were exercise-induced ischaemia or haemodynamic compromise, NYHA class III–IV symptoms, and significant limiting comorbidities, e.g., musculoskeletal, that would prevent full participation. Participant flow through the study, including eligibility screening, exclusions, enrollment, and completion of the 12 week training program, is illustrated in Figure 1.

### 2.2. Intervention and Testing Structure

**Figure 2:**
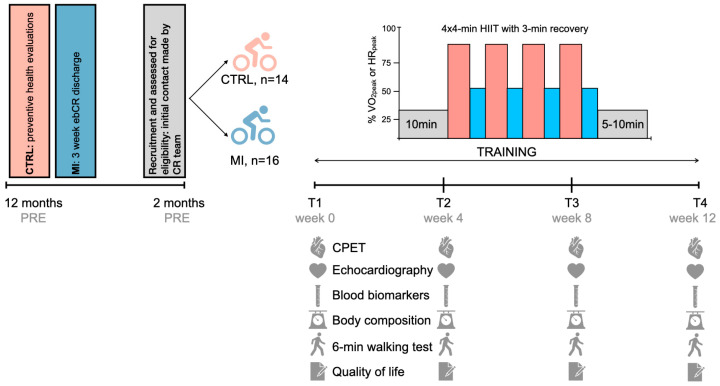
Study design, training protocol, and timeline of assessments.

Both MI and CTRL participants were engaged in an outpatient high-intensity interval training (HIIT) protocol performed three times per week for 12 weeks, in accordance with the European Society of Cardiology (ESC) Guidelines for ebCR [17]. Each session followed the standardized 4 × 4 min interval model, consisting of a 10 min warm up at low intensity. Each 4 min interval was performed at 85–90% of peak heart rate (HRpeak) or ~80–90% of VO_2_peak or RPE 14–16 corresponding to the high-intensity zone recommended by ESC for improving cardiorespiratory fitness in cardiac patients [17]. Training intensity was prescribed using a combined CPET-derived approach. Each interval was separated by 3 min of active recovery. Cool down consisted of 5–10 min of low-intensity cycling. The high-intensity intervals, as well as warm up, recovery and rest phases were prescribed according to CPET-derived HRpeak and the corresponding cycling workload obtained during the ramp test. Because heart-rate responses may be attenuated by beta-blocker therapy, particularly in the MI group, heart rate was not used in isolation; training intensity was additionally guided by external workload, Borg RPE (6–20) and continuous heart-rate telemetry. Participants underwent comprehensive functional and physiological assessments at four time points: T1 (week 0, baseline), T2 (week 4), T3 (week 8) and T4 (week 12, end of intervention). Testing procedures included cardiopulmonary exercise test (CPET), echocardiography, extensive biochemical blood analyses, body composition evaluation, functional testing and health-related quality of life (HRQoL) assessment using the RAND 36, described in detail in the following sections. The intensity of intervals was reviewed after the 4- and 8-week CPET assessments and recalibrated using the most recent CPET-derived HRpeak and workload, while maintaining the same relative intensity range and safety-monitoring procedures. This recalibration was performed to preserve the intended relative HIIT stimulus as exercise capacity improved during the intervention. The complete study design is shown in Figure 2.

Stable post–STEMI patients who had completed a standardized 3-week in-patient ebCR program approximately 12 months prior to enrollment, and healthy, previously inactive control participants (CTRL) without known cardiovascular, metabolic, or systemic disease, were recruited following eligibility screening by the cardiac rehabilitation team. Both groups completed a supervised 12-week high-intensity interval training (HIIT) program consisting of 4 × 4 min intervals at 85–90% HRpeak, ~80–90% of VO_2_peak, interspersed with 3 min active recovery, preceded by a standardized warm up and followed by a cool down. Comprehensive assessments were performed at baseline (T1, week 0), and after 4 (T2), 8 (T3), and 12 weeks (T4), including cardiopulmonary exercise testing (CPET), transthoracic echocardiography, blood biomarkers, body composition analysis, six-minute walking test (6MWT), and health-related quality of life evaluation.

### 2.3. Outcome Measures

#### 2.3.1. Cardiopulmonary Exercise Test (CPET)

A graded cardiopulmonary exercise test (CPET) was performed to assess the aerobic capacity of all participants. CPET was conducted on an electromagnetically braked cycle ergometer (Ergoline Ergoselect 5, GmbH, Bitz, Germany). The protocol consisted of 2 min of rest followed by 4 min of unloaded pedaling at a constant cadence between 55 and 65 rpm. Subsequently, the workload was set at 20 W and increased linearly by 10 W every minute until volitional exhaustion. This protocol was selected as a conservative standardized protocol suitable for clinically stable post-MI participants and previously inactive controls. The same ramp rate was used at all repeated assessments to preserve within-subject comparability across time points. Throughout the test, patients were continuously monitored using a 12-lead electrocardiogram (Quark C12X, Cosmed, Rome, Italy) and a pulse oximeter (OxyTrue, Bluepoint MEDICAL, Las Vegas, NV, USA). Gas-exchange variables—oxygen uptake (VO_2_), carbon dioxide output (VCO_2_), and minute ventilation (VE)—were collected breath-by-breath using an open-circuit metabolic cart (Quark CPET, Cosmed, Italy). Calibration of the flow turbine, volume sensors, and gas analyzers was performed prior to each test according to manufacturer specifications to ensure accuracy and reproducibility of measurements. Cardiorespiratory parameters (VO_2_, VCO_2_, VE, HR) and external power output (PO) were determined at the first ventilatory threshold (AT), the second ventilatory threshold (RCP) and at peak exercise. Peak values were defined as the mean of the final 20 s of the incremental phase. CPET was performed as a symptom-limited maximal or near-maximal exercise test. Test validity was assessed using an integrated approach including peak RER, perceived exertion, symptoms, cadence maintenance, and clinical safety criteria. Maximal or near-maximal effort was supported by a peak RER ≥ 1.10 and/or Borg RPE ≥ 17 on the 6–20 scale, together with volitional exhaustion, limiting symptoms, or inability to maintain the required cycling cadence of 60 rpm despite standardized verbal encouragement. AT and RCP were identified by two independent experienced evaluators, blinded to group and time point, using standardized gas-exchange criteria described in classical foundational work [18] and validated in clinical cardiopulmonary assessment research [19]. In the event of any discrepancy between their determinations, a third operator reviewed the data and provided the final adjudication.

#### 2.3.2. Transthoracic Echocardiography

Transthoracic echocardiography was performed to assess left ventricular structure and function in accordance with existing guidelines [20,21]. All examinations were conducted by an experienced cardiologist blinded to group assignment and time point. Imaging was obtained at rest using a commercially available ultrasound system (Vivid 9, GE Healthcare, Chicago, IL, USA), following standard acquisition procedures for evaluation of cardiac morphology and systolic and diastolic function [22]. The primary outcome, left ventricular systolic function, was assessed with particular emphasis on left ventricular ejection fraction (LVEF), which was quantified using the biplane Simpson method and served as the primary index of systolic performance. Left ventricular structural assessment included measurement of left ventricular end-diastolic diameter (LVEDd), interventricular septal thickness (IVS), and posterior wall thickness (PW). Diastolic function was evaluated using transmitral Doppler indices, including early (E) and late (A) diastolic inflow velocities, the E/A ratio and E/e′. Left atrial linear dimensions were recorded in the anteroposterior and transverse planes.

#### 2.3.3. Biochemical Analyses

Venous blood samples were collected in the morning on a rest day following an overnight fast and in accordance with routine clinical procedures. Samples were obtained at all four assessment time points (T1 to T4). Standardized laboratory analysis included a comprehensive cardiovascular and metabolic biomarker panel. Primary biochemical variables comprised triglycerides (mmol·L^−1^), total cholesterol (mmol·L^−1^), high-density lipoprotein (HDL, mmol·L^−1^) cholesterol, low-density lipoprotein (LDL, mmol·L^−1^) cholesterol, and derived lipid ratios (HDL/total cholesterol and LDL/HDL). In addition, N-terminal pro-B-type natriuretic peptide (NT-proBNP, pg·mL^−1^) was measured as a marker of cardiac stress and ventricular function. To assess systemic inflammation and subclinical immune activation, C-reactive protein (CRP, mg·L^−1^) was analyzed. High-sensitivity cardiac troponin (hs-troponin, ng·L^−1^) was included as a marker of myocardial injury and to ensure safety monitoring throughout the high-intensity exercise intervention. Endocrine status was evaluated via thyroid-stimulating hormone (TSH, mlU·L^−1^) and free thyroid hormones (fT3, fT4, pmol·L^−1^). Renal function was assessed using serum creatinine and the corresponding estimated glomerular filtration rate (eGFR, mL·min^−1^·1.73 m^−2^), calculated according to current laboratory standards.

#### 2.3.4. Anthropometric and Body-Composition Measurements

Body composition was assessed at admission and discharge using a calibrated Tanita bioelectrical impedance analyser (Tanita MC-780U Plus P, TANITA CORP, Arlington Heights, IL, USA) providing: body weight (kg),body mass index (BMI), body fat percentage (%) and absolute fat mass (kg), fat-free mass (kg) and muscle mass (kg), skeletal muscle mass (kg), basal metabolic rate (kcal), visceral fat index, waist-to-hip ratio (WHR). Standing height was measured using a Leicester Height Measure (SECA, Birmingham, UK). Waist circumference was obtained at 1 cm above the iliac crest, and hip circumference at the widest portion of the buttocks, in accordance with anthropometric guidelines [23]. Data were recorded using coded participant identifiers, and outcome extraction was performed without access to the longitudinal statistical results.

#### 2.3.5. Functional Assessment: Six-Minute Walk Test (6MWT)

Functional capacity was also assessed through the Six-Minute Walk Test (6MWT). It was performed according to the standard protocol recommended by the American Thoracic Society [24]. Briefly, the 6MWT test was conducted in a 60 m hallway marked every 5 m with tapes on the floor.

#### 2.3.6. Health-Related Quality of Life (HRQoL)

RAND-36 questionnaire was used as a validated measure of HRQoL [25]. The measure comprises eight dimensions: physical functioning, role limitations due to physical health, role limitations due to emotional problems, energy/fatigue, emotional well-being, social functioning, pain and general health. Responses to the RAND-36 questionnaire were scored according to standard RAND scoring procedures. Item responses were recoded so that higher scores reflected better health status and transformed to a 0–100 scale, with scores representing the percentage of the maximum possible value. Domain scores were calculated by averaging the relevant items within each of the eight scales.

### 2.4. Time Frame and Study Design

Two groups of participants participated in this 12-week observational study: (1) MI group; patients who had completed an in-patient cardiac rehabilitation and were subsequently included in an outpatient ebCR program, and (2) CTRL group of previously inactive participants without known cardiovascular, metabolic, or systemic disease. The study was conducted at the Hospital for Medical Rehabilitation of Heart and Lung Diseases and Rheumatism Thalassotherapia-Opatija (Croatia). The study protocol was approved by the institutional ethics committee of the hospital itself (reference number 01-000-00-216/2-2021) and was conducted in accordance with the principles of the Declaration of Helsinki. All participants provided written informed consent prior to enrollment.

### 2.5. Statistical Analysis

Data were analyzed using JASP (version 0.19.3 for macOS) and GraphPad Prism (Version 10.6.1 for macOS). Missing data were assessed before analysis. Participants with incomplete longitudinal follow-up or insufficient adherence were excluded from the final longitudinal analysis. For the analyzed cohort, analyses were performed using complete cases for each outcome. Baseline characteristics did not significantly differ between completers and non-completers across available variables. The sample size was determined by the feasibility of recruiting eligible participants and completing repeated multidomain assessments within the outpatient cardiac rehabilitation setting. Continuous variables are presented as median (IQR, interquartile range) unless otherwise stated. Normality of distributions was initially assessed using the Shapiro–Wilk test. Within-group changes across time (T1–T4) were assessed separately for each group using Friedman’s test. For outcomes with a significant Friedman test, post hoc pairwise comparisons were performed using Dunn’s test with Holm–Bonferroni adjustment for multiple comparisons. Effect size for significant Friedman tests was quantified using Kendall’s W (interpreted as small ≈ 0.10, moderate ≈ 0.30, and large ≥ 0.50). Between-group differences at each time point were analyzed using Mann–Whitney U tests. To control the family-wise error rate across repeated time-point comparisons within each outcome, *p*-values were adjusted using the Holm–Bonferroni method. Effect sizes for between-group comparisons are reported as rank-biserial correlations with 95% confidence intervals. Moreover, exploratory linear mixed-effects models were performed for the primary and selected key secondary outcomes to formally assess longitudinal group × time interactions. Models included group, time point, and group × time interaction as fixed effects, participant ID as a random intercept, and adjustment for age, baseline VO_2_peak, and baseline LVEF. Model-adjusted estimated marginal means with 95% confidence intervals were reported. All statistical tests were two-sided, and statistical significance was set at *p* < 0.05.

## 3. Results

Outcomes were organized hierarchically for clarity. VO_2_peak and LVEF were defined as primary endpoints. Key secondary endpoints included VO_2_ at AT and RCP, LVEDd, NT-proBNP, hs-troponin, CRP, 6MWT distance, and selected body-composition outcomes, including body weight, body fat percentage, and muscle mass. All remaining cardiopulmonary, echocardiographic, biochemical, body-composition, and HRQoL variables were considered exploratory and are reported in Supplementary Table S1.

All participants included in the final longitudinal analysis completed 36 supervised HIIT sessions. Missed sessions were rescheduled within the intervention period when possible; participants who missed one full training week or discontinued the intervention were excluded from the longitudinal analysis. No clinically relevant adverse events were recorded during the supervised HIIT intervention. The study included stable inactive post-STEMI patients (MI group; age 57.6 ± 10.2 years; height 177 ± 9.5 cm; body weight 86 ± 15.3 kg, 15 males, 1 female) and previously inactive participants without known cardiovascular, metabolic or systemic disease (CTRL; age 48.6 ± 10.7 years; height 175 ± 13.7 cm; body weight 86 ± 18.0 kg, 12 males, 2 females). In the MI group, standard secondary-prevention pharmacological therapy was maintained without modification during the 12-week intervention period. All MI participants were clinically stable during the pre-enrollment period and throughout the 12-week intervention, with no documented recurrent myocardial infarction, hospitalization for heart failure, unstable arrhythmias, or exercise-limiting cardiac symptoms before study inclusion. Detailed baseline clinical characteristics of the MI group, including infarct-related variables, revascularization status, prior in-patient ebCR exposure, cardiovascular risk factors and medication exposure, are presented in Table 1. Time from index STEMI to baseline HIIT enrollment was not significantly correlated with the change in LVEF from T1 to T4 (Spearman ρ = −0.261, *p* = 0.330) or with the change in VO_2_peak from T1 to T4 (ρ = 0.044, *p* = 0.871).

### 3.1. Cardiopulmonary Exercise Test (CPET)

**Figure 3:**
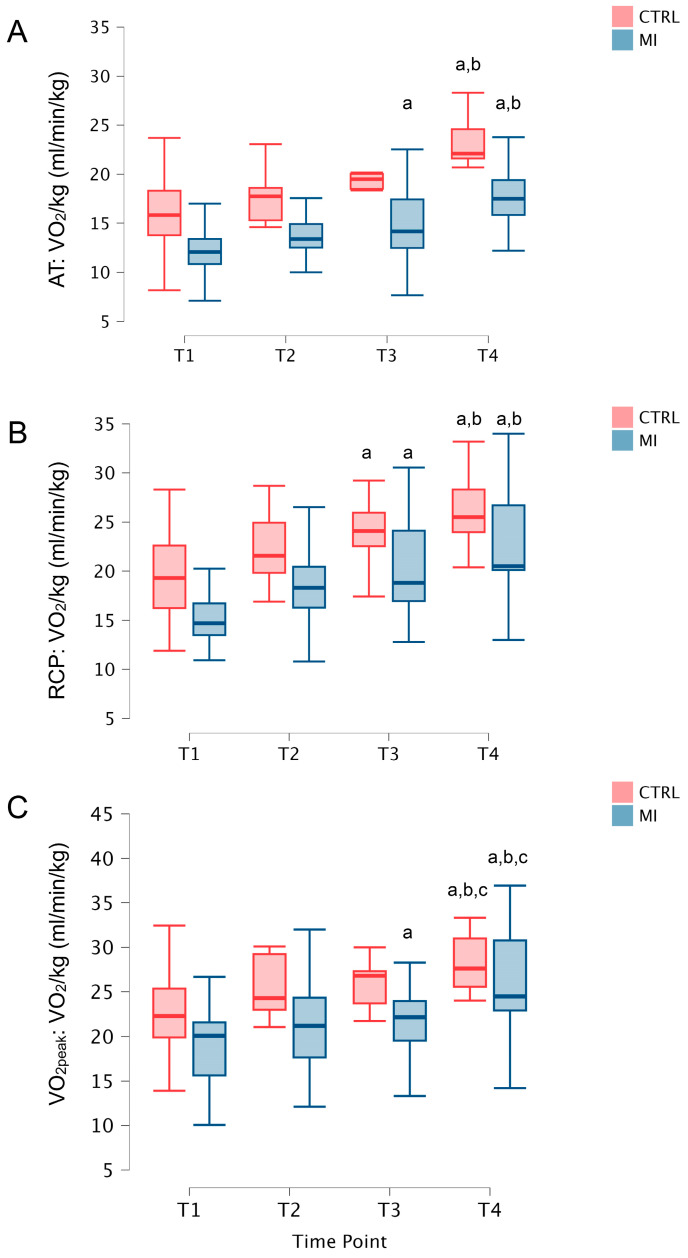
Time-course of ventilatory thresholds and peak oxygen uptake during the 12-week HIIT intervention. Box-and-whisker plots illustrating changes in (**A**) first ventilatory threshold (AT), (**B**) second ventilatory threshold (RCP), and (**C**) peak oxygen uptake (VO_2_peak) across four assessment time points (T1–T4) in MI and CTRL. Data are presented as median (central line), interquartile range (box), and range (whiskers). Superscript letters indicate significant within-group pairwise differences (a vs. T1; b vs. T2; c vs. T3). Between-group differences were assessed at each time point using non-parametric tests.

In the MI group, VO_2_ at AT increased significantly over time (Friedman χ^2^(3) = 39.72, *p* < 0.001), with post hoc comparisons showing higher values at T3 and T4 compared with T1 and additional increases at T4 compared with T2 (Table 2, Figure 3A). Similarly, VO_2_ at RCP demonstrated a significant temporal increase in the MI group (χ^2^(3) = 40.81, *p* < 0.001), with the first significant improvement at T3 compared with T1, and at T4 compared with T1 and T2. In the CTRL group, both AT and RCP VO_2_ increased over time (AT: χ^2^(3) = 23.40, *p* < 0.001; RCP: χ^2^(3) = 28.89, *p* < 0.001), with significant pairwise increases observed at T4 compared with earlier time points (Table 2, Figure 3B).

VO_2_peak increased significantly in the MI group (χ^2^(3) = 39.60, *p* < 0.001). Post hoc analysis indicated higher values at T3 compared with T1, and at T4 compared with T1, T2, and T3. In the CTRL group, VO_2_peak also showed a significant overall time effect (χ^2^(3) = 27.69, *p* < 0.001), with a significant increase only at T4 (Table 2, Figure 3C).

Between-group comparisons showed no significant differences in VO_2_-derived CPET parameters at any time point after Holm adjustment; however, heart rate responses at AT, RCP and peak exercise were consistently higher in the CTRL group across all assessments (Table 3).

### 3.2. Transthoracic Echocardiography

**Figure 4:**
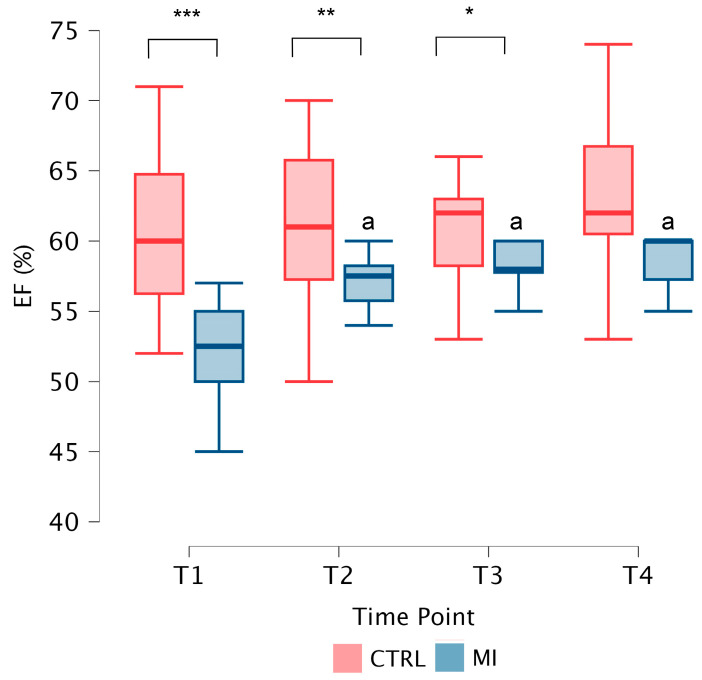
Changes in left ventricular ejection fraction during the 12-week HIIT intervention. LVEF at baseline (T1) and after 4 (T2), 8 (T3), and 12 weeks (T4) of HIIT in MI and CTRL groups. Data are presented as median (horizontal line), interquartile range (box), and range (whiskers). Superscript letters indicate significant within-group pairwise differences (a vs. T1; b vs. T2; c vs. T3). Asterisks denote significant between-group differences at the corresponding time point (* *p* < 0.05, ** *p* < 0.01, *** *p* < 0.001).

Within the MI group, LVEF improved significantly over time (χ^2^(3) = 36.52, *p* < 0.001), increasing from 52.5% (50.0–55.0) at baseline to 60% (55.8–60.0) at T4, with post hoc analyses indicating higher LVEF at T2, T3 and T4 compared with T1, without additional pairwise differences between subsequent time points (Figure 4). No significant temporal change in LVEF was observed in the CTRL group (χ^2^(3) = 4.60, *p* = 0.20). LV volumes and GLS were not available in this group. LVEDd and PW remained stable over time in both groups. Although IVS showed a significant overall time effect, post hoc comparisons did not indicate a clear pattern of change (Supplementary Table S1). In the CTRL group, the E/A ratio increased significantly at T4 compared with T1 and T2 (χ^2^(3) = 23.23, *p* < 0.001; Supplementary Table S1). Between-group comparisons revealed lower LVEF in the MI group at T1, T2 and T3; however, this difference was no longer statistically significant at T4 following Holm adjustment (Figure 4, Table 3). The MI group also exhibited higher E/e′ at baseline and early time points, but these differences did not persist at later assessments (Supplementary Table S2).

### 3.3. Biochemical Analyses

Triglycerides did not exhibit significant longitudinal changes in either group after post hoc adjustment. In the CTRL group, LDL cholesterol and LDL/HDL ratio decreased significantly over time (LDL: χ^2^(3) = 14.65, *p* = 0.002; LDL/HDL: χ^2^(3) = 10.49, *p* = 0.015), whereas total cholesterol and HDL did not demonstrate significant pairwise changes after correction (Supplementary Table S1). Within the MI group, no significant changes over time were observed in total cholesterol, LDL, HDL, or HDL/CHOL ratio.

CRP decreased significantly in the MI group (χ^2^(3) = 11.80, *p* = 0.008), with a reduction evident between T1 (2.1 mg·L^−1^ (0.4–3.1)) to T4 (0.7 mg·L^−1^ (0.2–1.7). NT-proBNP did not significantly change over time in either group (MI: χ^2^(3) = 2.31, *p* = 0.510; CTRL: χ^2^(3) = 2.29, *p* = 0.515; Table 2). In contrast, hs-troponin increased significantly over time in the MI group, from 8.0 ng·L^−1^ (6.3–9.4) at T1 to 10.5 ng·L^−1^ (8.4–12.6) at T4 (χ^2^(3) = 25.38, *p* < 0.001). Although statistically significant, the absolute increase was small and values remained relatively low. In the CTRL group, hs-troponin also showed a significant time effect (χ^2^(3) = 11.23, *p* = 0.011), but values did not show a progressive increase, changing from 5.0 ng·L^−1^ (4.7–7.3) at T1 to 4.9 ng·L^−1^ (4.3–6.8) at T4, with the only significant post hoc difference observed between T2 and T4. These longitudinal biochemical changes are summarized in Table 2 and detailed in Supplementary Table S1.

Between-group comparisons confirmed persistently higher NT-proBNP and hs-troponin concentrations in the MI group across all time points. Conversely, total cholesterol, LDL cholesterol, and LDL/HDL ratio were consistently higher in the CTRL group (Supplementary Table S2).

### 3.4. Body Composition

In the MI group, body weight did not change significantly over time, whereas body fat percentage decreased (χ^2^(3) = 18.27, *p* < 0.001) from 25.7% (24.1–30.8) at T1 to 24.8% (23.4–29.1) at T4. Muscle mass remained unchanged. In the CTRL group, both body weight and body fat percentage decreased significantly over time (weight: χ^2^(3) = 15.69, *p* = 0.001; fat %: χ^2^(3) = 20.74, *p* < 0.001), while muscle mass and visceral fat remained unchanged (Table 2). Between-group differences were not significant at any time point after Holm correction (all *p* > 0.05; Table 3).

### 3.5. Functional Assessment: Six-Minute Walk Test (6MWT)

Both groups demonstrated significant improvements in functional capacity, as measured by distance walked during the 6MWT (MI: χ^2^(3) = 32.81, *p* < 0.001; CTRL: χ^2^(3) = 18.70, *p* < 0.001). No significant between-group differences were observed after Holm adjustment (Table 2).

### 3.6. Health-Related Quality of Life (HRQoL)

No significant longitudinal changes in any RAND-36 domain were observed in the MI group. In the CTRL group, general health showed a significant improvement at T3 compared with baseline, while other domains did not show significant pairwise changes (Supplementary Table S1). Between-group comparisons revealed lower physical functioning scores in the MI group at T2–T4 and lower general health scores at all time points after Holm correction. A baseline between-group difference was also present for social functioning (Supplementary Table S2).

### 3.7. Effect Size for Longitudinal Changes

The magnitude of within-group time effects ranged from small to strong for all described outcomes, as indicated by Kendall’s coefficient of concordance (W), reported in Supplementary Table S1.

### 3.8. Linear Mixed-Effects Analyses

Linear mixed-effects analyses were performed for the primary outcomes and selected key secondary outcomes, adjusted for age, baseline VO_2_peak, and baseline LVEF and results are presented as model-adjusted estimated marginal means with 95% confidence intervals. VO_2_peak increased over time in both groups, from 20.76 (95% CI 19.12–22.40) to 27.07 (95% CI 25.43–28.71) mL·kg^−1^·min^−1^ in CTRL and from 21.66 (95% CI 20.15–23.16) to 28.02 (95% CI 26.51–29.52) mL·kg^−1^·min^−1^ in MI, without a significant group × time interaction (p_interaction_ = 0.684). LVEF showed a significant group × time interaction (p_interaction_ = 0.020), increasing from 57.92% (95% CI 56.11–59.74) to 60.42% (95% CI 58.61–62.24) in CTRL and from 54.51% (95% CI 52.83–56.18) to 60.88% (95% CI 59.20–62.56) in MI. No significant group × time interactions were observed for LVEDd, NT-proBNP, body weight, body fat, muscle mass, or 6MWT (all p_interaction_ ≥ 0.055), whereas significant group × time interaction was observed for CRP (p_interaction_ = 0.026) and hs-troponin (p_interaction_ < 0.001).

## 4. Discussion

The present study provides a descriptive longitudinal characterization of changes in exercise capacity, cardiac function, and cardiac stress biomarkers during a 12-week supervised HIIT-based outpatient ebCR program in patients with prior MI, compared with a previously inactive participants without known cardiovascular, metabolic or systemic disease.

The main findings were that VO_2_peak improved significantly over time in both groups, while LVEF improved more prominently in the MI group. Exploratory linear mixed-effects sensitivity analyses supported these findings by showing no significant group × time interaction for VO_2_peak, suggesting comparable adjusted training-related trajectories between groups, whereas LVEF showed a significant group × time interaction, indicating a larger adjusted improvement in MI. NT-proBNP remained stable, while CRP and hs-troponin showed exploratory group × time interactions that require cautious interpretation.

First, both groups demonstrated significant within-group time effects for VO_2_-derived CPET parameters. However, the temporal pattern of pairwise improvements differed. In the MI group, significant increases in VO_2_ at AT, RCP, and VO_2_peak were evident from T3 onward, suggesting an earlier onset of measurable adaptation. In contrast, in the CTRL group, significant pairwise differences were observed predominantly at T4, suggesting a later manifestation of training-related improvements.

Second, LVEF increased significantly over time only in the MI group (from 4 weeks onward); early between-group differences in LVEF and E/e′ were present but were no longer significant at later assessments. Third, NT-proBNP concentrations did not change longitudinally within either group but remained consistently higher in the MI group across all time points, suggesting persistent differences in cardiac stress despite functional and echocardiographic improvement. The longitudinal increase in hs-troponin observed in the MI group requires cautious interpretation. Although the change reached statistical significance, the absolute hs-troponin concentrations remained relatively low. Importantly, NT-proBNP did not show a statistically significant longitudinal change during the supervised intervention, despite minor numerical fluctuations across time points. Therefore, the hs-troponin finding should be interpreted cautiously and should not be considered, on its own, as evidence of clinically significant myocardial injury. Rather, it may reflect a combination of physiological exercise-induced troponin release, biological and analytical assay variability, and transient myocardial stress responses associated with repeated high-intensity exercise exposure.

Exercise-induced increases in cardiac troponin have been described after different forms of physical exercise, including in individuals without overt cardiovascular disease, and are often characterized by an early post-exercise rise followed by a decline within approximately 24 h. However, in post-MI patients, even small changes in hs-troponin require careful clinical interpretation because baseline myocardial vulnerability, prior infarction, medication use, and individual training responses may influence biomarker dynamics. The present study was not designed to determine the mechanism of hs-troponin release, and blood samples were not collected immediately before and after individual HIIT sessions. Consequently, it remains unclear whether the observed increase represents a benign physiological adaptation, assay-related variability, or a signal of repeated transient myocardial stress. Future studies should include session-specific pre- and post-exercise hs-troponin sampling, longer follow-up, and integration with ECG, echocardiographic, and clinical outcomes to clarify the clinical relevance of this finding.

The present findings should be interpreted in the context of prior HIIT-based cardiac rehabilitation evidence. The novelty of the present study is not repeated assessment alone, but the combined 4-weekly evaluation of CPET-derived indices, echocardiographic function, biomarkers, body composition, functional capacity, and HRQoL within the same supervised HIIT-based outpatient ebCR program. This multidomain design allowed descriptive characterization of how different clinically relevant outcomes changed over time in carefully selected clinically stable low-risk post-STEMI patients, while remaining hypothesis-generating rather than causal or comparative.

The observed pattern of CPET adaptations underscores the value of repeated intermediate assessments during ebCR. In the MI group, significant improvements in AT and RCP coincided with increases in VO_2_peak, suggesting a pattern consistent with synchronous adaptation of submaximal and maximal exercise capacity during HIIT-based rehabilitation. This is in contrast to what was shown in a cohort of older but healthy participants, where improvements in AT occurred before increases in VO_2_peak [10], a pattern that has been attributed to early peripheral adaptations, including mitochondrial remodeling, although such mechanisms were not directly assessed in the present study. Central cardiovascular adaptations (i.e., blood volume and cardiac output), which are thought to contribute to HIIT-induced increases in VO_2_peak, may develop progressively over the course of a training program [5]. Our observed ~22% increase in VO_2_peak, in the MI group is consistent with the literature, where supervised cardiac rehabilitation in post-acute coronary syndromes patients produces clinically meaningful increases in cardiorespiratory fitness; for example, one cohort analysis found that over 30% of patients achieved ≥ 15% VO_2_peak, improvement after completion of rehabilitation, with greater training exposure associated with larger gains [26]. However, the linear mixed-effects sensitivity analysis did not show a significant group × time interaction for VO_2_peak. Therefore, these findings should be interpreted as evidence of substantial improvement in both groups rather than as proof of a distinct or earlier MI-specific trajectory. The comparable adjusted VO_2_peak response also suggests that carefully selected, clinically stable post-MI patients retained the capacity for robust cardiorespiratory adaptation during supervised HIIT-based ebCR.

In the MI group, improvements in VO_2_/WR slope and VO_2_/HR paralleled significant increases in AT, RCP and VO_2_peak over the 12-week HIIT program, which may reflect enhanced aerobic efficiency and stroke volume response during exercise. VO_2_/WR slope reflects the oxygen cost for a given work rate and is interpreted as a marker of integrated cardiovascular and peripheral efficiency; improvements are consistent with both central (cardiac output) and peripheral (muscle oxygen utilization) adaptations occurred with training [27]. Similarly, changes in VO_2_/HR have been associated with differences in cardiac performance after MI, with variations in oxygen pulse reflecting potential alterations in stroke volume and cardiac function, supporting its complementary value alongside VO_2_peak [28]. Whereas VO_2_/WR slope and VO_2_/HR responses have been described in heart failure and chronic cardiovascular disease as markers of underlying pathophysiology [29] our results extend these observations by showing improvements in these dynamic CPET indices following training, alongside concurrent gains in VO_2_peak and ventilatory threshold parameters in post-MI patients.

Findings from this study suggest that HIIT-based ebCR was associated with early (4 weeks) improvement of systolic function in stable post-MI patients. The absence of LVEF change in CTRL is consistent with the interpretation that the observed change in MI may be consistent with functional recovery of previously injured myocardium [30] rather than physiological variability. In line with this, LVEF demonstrated a significant group × time interaction, supporting a more pronounced improvement in systolic function in the MI group.

Previous studies in post-MI and CAD populations have reported improvements in LVEF following structured exercise training, including HIIT-based interventions, although most relied on pre-post designs without intermediate assessment [12,31]. Giallauria et al. demonstrated that early initiation of exercise training after ST-elevation MI was associated with an improvement in LV function [31], likely due to the improvement in resting and post-stress LV wall motion and thickening score indexes and ejection fraction and with improvements in cardiovascular functional capacity. An important contribution of the present study is the repeated assessment of LVEF and CPET-derived indices. In this cohort, LVEF improved by T2, whereas some efficiency-related CPET indices showed significant changes later in the program. However, given the descriptive design and limited sample size, these findings should not be interpreted as evidence of a mechanistic sequence of adaptation, but rather as hypothesis-generating observations requiring confirmation in larger studies.

LVEDd remained stable in the MI group, while a small increase was observed in CTRL. Importantly, the linear mixed-effects sensitivity analysis did not show a significant group × time interaction for LVEDd, supporting the absence of adverse left ventricular dilatation in the MI cohort. These findings are consistent with structural stability rather than ongoing remodeling in this contemporary post-MI population [32].

LVEF demonstrated statistically significant within-group increases in the MI cohort, as indicated by time-point-specific differences relative to baseline, whereas it remained unchanged in controls. Importantly, these changes occurred in the absence of parallel alterations in left ventricular dimensions or wall thickness, supporting a functional rather than structural mechanism potentially underlying LVEF improvement. Such dissociation between LVEF and chamber geometry has been described in post-MI patients receiving optimized therapy and rehabilitation [33].

E/A ratio significantly increased in CTRL group over time. Given that control group was slightly younger than MI patients and free of overt cardiovascular disease, this increase is most consistent with physiological modulation of diastolic filling related to loading conditions, autonomic balance, or training-related effects rather than pathological diastolic progression. Importantly, interpretation of isolated E/A changes should be cautious, as current echocardiographic guidelines emphasize the limited specificity of transmitral inflow indices when assessed without complementary tissue Doppler or structural markers [20].

E/e′ ratios differed significantly between groups at T1 and T2, with higher values in the MI group compared with controls, while no within-group changes were observed across time in either cohort. Absolute E/e′ values remained in a range generally not consistent with elevated left ventricular filling pressures according to echocardiographic guideline criteria (average E/e′ > 14), suggesting that the between-group difference may reflect subtle alterations in relaxation/compliance rather than overt elevation of filling pressures [20].

Several statistically significant findings among secondary exploratory outcomes, including small changes in IVS and selected endocrine markers, should be interpreted cautiously. Although these outcomes reached statistical significance, the absolute magnitude of change was small and may not represent clinically meaningful adaptation.

In stable post-STEMI populations with preserved systolic function, NT-proBNP is primarily a prognostic marker rather than a training-responsive biomarker, and its stability following cardiac rehabilitation may be interpreted as a reassuring indicator of safety for future risk stratification [34]. The stable response observed in our study is consistent with evidence supporting the safety of supervised HIIT in coronary artery disease, with low adverse event rates when appropriately prescribed [35]. Although hs-troponin slightly increased in MI group, it remained below assay-specific 99th-percentile upper reference limits, supporting interpretation as low-grade myocardial stress or exercise-related myocardial turnover rather than acute ischemic injury [36]. Conversely, CRP declined over time (from a median of 2.1 in T1 to 0.7 in T4, −66.7%), consistent with the anti-inflammatory effects of structured exercise training observed in CR [37]. Total cholesterol and LDL were higher in controls throughout follow-up, most plausibly reflecting differences in secondary-prevention lipid-lowering therapy in MI group, particularly given that no structured nutritional counseling was provided during the intervention [38].

Most body-composition variables remained stable, although body fat percentage decreased modestly in both groups. The modest but statistically significant reductions in body fat percentage observed in T4 for both groups align with prior studies demonstrating that CR typically produces small-to-moderate reductions in adiposity rather than substantial weight loss, even during longer programmes [39]. Moreover, individualized nutritional counseling was not provided. It is plausible to expect that more improvements might have been observed if ebCR was combined with structured behavioral weight-loss interventions [40]. A positive finding was the stability of muscle mass throughout the training program in both groups, even though no specific resistance training outside of HIIT was performed.

Submaximal functional capacity, assessed by the 6MWT, improved significantly over time in both MI and control groups. The absence of between-group differences suggests that MI group retained the ability to respond to ebCR, achieving improvements comparable to those observed in controls.

The difference between groups and the lack of improvement in HRQoL outcomes is consistent with prior ebCR literature showing that improvements in objective measures of physical capacity are more consistently observed than changes in HRQoL, particularly in patients with established CAD. However, changes in HRQoL outcomes after ebCR are often modest; a Cochrane CHD review noted that even if HRQoL improvements are detected, that may not be clinically important [41]. It is important to note that the relatively small number of participants may also explain the lack of statistical significance.

Finally, secondary-prevention pharmacotherapy should be considered as a potential biological confounder influencing cardiometabolic and functional recovery after MI. In the present cohort, beta-blockers may have contributed to the lower heart-rate responses observed in the MI group, while statins and other secondary-prevention therapies may have influenced lipid profile, inflammatory status, ventricular function, and biomarker trajectories. SGLT2 inhibitors and GLP-1 receptor agonists are increasingly discussed in contemporary ACS populations because of their potential cardiometabolic benefits; however, no MI participant in the present cohort was treated with either drug class. Therefore, these agents did not contribute to confounding in this study, although their absence should be considered when comparing our findings with future cohorts treated with broader contemporary cardiometabolic therapy [42,43].

### Limitations and Strengths

Several limitations and strengths should be acknowledged when interpreting the findings of this study. First, the relatively small sample size and the large number of investigated outcomes limit statistical power and increase the possibility of type I error. Therefore, the findings should be interpreted as exploratory and hypothesis-generating. Future larger, ideally multi-center studies are necessary to validate and extend the present findings. Second, the control group consisted of participants without known cardiovascular, metabolic or systemic disease that were previously inactive rather than a non-exercising MI control group, which, while allowing contextualization of training responses against a physically comparable reference group, further limits the ability to distinguish post-MI-specific adaptations from general training effects. Therefore, the observed improvements should be interpreted as longitudinal adaptations during supervised HIIT-based ebCR, rather than evidence of superiority or specificity compared with other rehabilitation modalities. Third, the inclusion of clinically stable, low-risk post-MI patients eligible for supervised HIIT restricts generalizability to higher-risk or unstable populations. The exclusion of patients receiving anticoagulant therapy further limits generalizability to broader post-MI populations, including patients with atrial fibrillation or other indications for long-term anticoagulation. The small proportion of female participants further limits the applicability of findings to women with prior myocardial infarction. Fourth, pharmacological therapy was not controlled beyond standard secondary prevention management, and medication effects may have influenced cardiac function, heart rate responses, and biomarker profiles. However, no changes in pharmacological therapy occurred during the 12-week intervention period, reducing the likelihood that medication adjustments confounded the observed adaptations. The interpretation of hs-troponin changes is further limited by the absence of session-specific pre- and post-exercise biomarker sampling. Therefore, we could not determine whether the observed longitudinal increase reflected transient exercise-induced troponin release, biological or analytical variability, or repeated subclinical myocardial stress. This issue should be addressed in future studies with more detailed biomarker kinetics and longer clinical follow-up.

Echocardiographic interpretation was also limited by the absence of LV volume data and GLS. Consequently, the observed increase in LVEF should be interpreted as a change in systolic functional measurement rather than evidence of structural reverse remodeling.

Although participants were enrolled in a clinically stable phase after completion of in-patient ebCR, the contribution of spontaneous late post-infarction remodeling cannot be fully excluded. Therefore, the observed LVEF changes should be interpreted cautiously as functional improvement during the rehabilitation period rather than definitive evidence of structural reverse remodeling.

Finally, although repeated assessments allowed descriptive evaluation of temporal patterns across domains, the study was not designed to test mechanistic sequencing of physiological adaptation. Therefore, observations suggesting non-parallel changes across CPET, echocardiographic, biomarker, functional, or HRQoL outcomes should be interpreted as exploratory and hypothesis-generating.

Because only participants who completed the full intervention were analyzed, the findings may reflect responses among adherent completers rather than all initially assessed participants. However, this complete-case approach ensured that longitudinal analyses were based on participants with fully observed repeated measurements across all four time points.

Despite these limitations, an important strength of this study is the incorporation of repeated intermediate assessments during a standardized HIIT-based ebCR program, enabling detailed characterization of the time course of adaptation across functional, cardiac, and biomarker domains rather than relying solely on pre-post comparisons. This multidimensional and temporally resolved design provides novel insight into the time course of physiological responses during post-MI rehabilitation.

## 5. Conclusions

This study provides a detailed longitudinal characterization of physiological, cardiac, biochemical, functional, and patient-reported adaptations during a 12-week supervised HIIT-based outpatient exercise-based cardiac rehabilitation program in carefully selected, stable, low-risk post-STEMI patients. During the intervention, the MI group demonstrated substantial improvements in cardiorespiratory fitness, including an approximately 22% increase in VO_2_peak, alongside early improvement in LVEF (~9.5% increase in 4 weeks) and stable NT-proBNP concentrations. Repeated intermediate assessments suggested that changes in CPET-derived indices, systolic function, biomarkers, functional capacity, and HRQoL did not occur in a uniform temporal pattern; however, these observations are descriptive and should not be interpreted as evidence of mechanistic sequencing. Larger randomized studies with true clinical comparator groups are required to determine the specificity and comparative effectiveness of HIIT-based cardiac rehabilitation after MI. Overall, these findings underscore the value of repeated multidomain assessment during cardiac rehabilitation and support further evaluation of supervised HIIT-based ebCR in larger randomized studies with appropriate clinical comparator groups.

## Figures and Tables

**Table 1:** Baseline clinical characteristics, treatment history, cardiovascular risk factors, and medication exposure in the MI group.

Variable	MI (*n* = 16)
Age (years, mean ± SD)	57.6 ± 10.2
Sex:	
Male (%)	15 (93.8)
Female (%)	1 (6.2)
Height (cm)	177 ± 9.5
Weight (kg)	86 ± 15.3
Timeline (mean ± SD)	
Days since STEMI	351 ± 157
Days since in-patient CR completion	198 ± 110
LVEF at in-patient CR discharge (mean ± SD)	47.3 ± 8.9
Treatment (*n*, %)	
Primary PCI for acute STEMI	12 (75.0)
Additional elective PCI after acute STEMI	2 (12.5)
CABG after acute STEMI due to multivessel coronary disease	3 (18.8)
Infarct-related artery, *n* (%)	
LAD	6 (37.5)
RCA	3 (18.8)
LCx	3 (18.8)
Multivessel/other	4 (25.0)
Medication	
Beta-blocker	14 (87.5)
Anti-hypertension	14 (87.5)
Anti-platelet	16 (100.0)
Anti-anginal	4 (25.0)
Statin	13 (81.3)
Diuretic	4 (25.0)
CVD risk factors	
Hypertension	10 (62.5)
Family history	6 (37.5)
Dyslipidemia	11 (68.8)
Mental health	3 (18.8)
Type II diabetes	0 (0.0)
Smoking	
Never	10 (62.5)
Former	4 (25.0)
Current	2 (12.5)
Alcohol	
Never	10 (62.5)
Occasionally	6 (37.5)
Excess	0 (0.0)

Values are presented as mean ± SD for continuous variables and *n* (%) for categorical variables. Percentages were calculated using the total MI cohort (*n* = 16). Days since STEMI and days since in-patient CR completion refer to the time from the index event or completion of the prior in-patient cardiac rehabilitation program to baseline enrollment in the present outpatient HIIT-based ebCR intervention. Infarct-related artery was defined according to the documented culprit artery on coronary angiography and/or STEMI localization in the clinical records. Revascularization categories are not mutually exclusive because patients may have undergone primary PCI for the acute STEMI followed by additional elective PCI or CABG due to multivessel coronary artery disease. Medication classes are reported according to documented baseline therapy. STEMI, ST-elevation myocardial infarction; CR, cardiac rehabilitation; HIIT, high-intensity interval training; ebCR, exercise-based cardiac rehabilitation; LVEF, left ventricular ejection fraction; PCI, percutaneous coronary intervention; CABG, coronary artery bypass grafting; CVD, cardiovascular disease; MI, myocardial infarction; SD, standard deviation. LAD: Left Anterior Descending Artery, RCA: Right Coronary Artery, LCx: Left Circumflex Artery.

**Table 2:** Primary and key secondary longitudinal outcomes across four time points (T1–T4) during the 12-week HIIT-based exercise-based cardiac rehabilitation intervention.

	Group	T1	T2	T3	T4	Friedman χ^2^(3)	*p*
Cardiopulmonary outcomes							
AT: VO_2_ (mL·kg^−1^·min^−1^)	MI	12.1 (10.7–13.6)	13.4 (12.4–15.5)	14.2 (12.2–17.8) ^a^	17.5 (15.5–20.5) ^a,b^	39.72	<0.001
	CTRL	15.9 (13.3–19.2)	17.8 (14.9–19.6)	19.5 (17.6–20.9)	22.1 (21.3–25.4) ^a,b^	23.4	<0.001
RCP: VO_2_ (mL·kg^−1^·min^−1^)	MI	14.6 (12.3–17.1)	18.2 (14.2–21.2)	18.4 (15.0–25.4) ^a^	20.5 (19.5–27.8) ^a,b^	40.81	<0.001
	CTRL	19.3 (15.9–23.6)	21.6 (19.3–25.2)	24.1 (21.9–26.8) ^a^	25.5 (23.8–28.5) ^a,b^	28.89	<0.001
VO_2_peak: VO_2_ (mL·kg^−1^·min^−1^)	MI	20.1 (15.6–22.7)	21.2 (16.9–25.1)	22.2 (19.5–25.3) ^a^	24.5 (22.7–32.3) ^a,b,c^	39.6	<0.001
	CTRL	22.3 (19.5–27.1)	24.3 (22.8–29.4)	26.8 (23.1–28.1)	27.6 (25.3–31.8) ^a,b,c^	27.69	<0.001
Echocardiographic outcomes							
LVEDd (mm)	MI	52.0 (47.3–55.0)	51.5 (48.3–55.0)	51.0 (48.0–54.5)	53.0 (50.5–55.0)	4.70	0.195
	CTRL	48.0 (46.3–50.8)	48.5 (47.3–51.5)	49.0 (44.5–51.0)	50.0 (47.5–51.3) ^a^	17.35	<0.001
LVEF (%)	MI	52.5 (50.0–55.0)	57.5 (55.2–58.7) ^a^	58.0 (57.2–60.0) ^a^	60.0 (55.8–60.0) ^a^	36.52	<0.001
	CTRL	60.0 (55.7–65.5)	61.0 (57.0–66.3)	62.0 (58.0–63.7)	62.0 (59.5–67.7)	4.6	0.200
Biochemical outcomes							
NT-proBNP (pg·mL^−1^)	MI	90.5 (81.2–227.3)	139.5 (64.8–263.8)	130.0 (53.3–282.5)	115.0 (68.5–206.8)	2.312	0.510
	CTRL	44.5 (20.8–50.5)	41.0 (25.0–73.8)	55.0 (29.8–64.8)	39.5 (21.0–63.5)	2.289	0.515
CRP (mg·L^−1^)	MI	2.1 (0.4–3.1)	1.3 (0.3–3.3)	1.0 (0.4–1.7)	0.7 (0.2–1.7) ^a^	11.80	0.008
	CTRL	1.2 (0.6–3.9)	1.0 (0.7–2.3)	2.9 (1.1–3.3)	1.2 (1.0–2.3)	6.088	0.107
hs-troponin (ng·L^−1^)	MI	8.0 (6.3–9.4)	8.9 (7.7–11.5) ^a^	10.2 (8.6–11.4) ^a^	10.5 (8.4–12.6) ^a^	25.38	<0.001
	CTRL	5.0 (4.7–7.3)	5.8 (5.4–6.9)	5.0 (4.8–6.9)	4.9 (4.3–6.8) ^b^	11.23	0.011
Body composition outcomes							
Body weight (kg)	MI	85.5 (79.0–89.9)	85.3 (78.2–88.8)	84.7 (77.7–90.3)	82.8 (76.9–90.5)	4.95	0.176
	CTRL	87.8 (66.9–103.2)	87.7 (67.5–102.9)	86.5 (66.2–100.5) ^a^	85.9 (66.1–99.2) ^a^	15.69	0.001
Body fat (%)	MI	25.7 (24.1–30.8)	25.7 (23.5–30.7)	25.8 (24.4–29.3)	24.80 (23.4–29.1) ^a^	18.27	<0.001
	CTRL	29.1 (22.6–33.5)	28.40 (22.2–33.4)	28.5 (20.8–31.8) ^a^	27.8 (22.5–32.8) ^a^	20.74	<0.001
Muscle mass (kg)	MI	61.3 (58.6–64.1)	61.4 (58.5–63.8)	61.8 (59.4–64.2)	61.1 (58.3–64.3)	0.375	0.945
	CTRL	61.2 (44.2–68.9)	62.5 (44.3–69.4)	62.8 (45.6–68.7)	62.6 (45.1–68.2)	6.8	0.079
Functional outcomes							
6MWT (m)	MI	675 (607.3–746.3)	721 (638.8–768.8) ^a^	712.5 (661.8–798.8) ^a^	745.0 (662.5–811.3) ^a^	32.81	<0.001
	CTRL	714 (630.0–800.0)	730.5 (665.0–827.8)	762.5 (666.3–828.8)	775.0 (688.8–861.8) ^a^	18.7	<0.001

Primary and key secondary longitudinal outcomes are summarized. VO_2_peak and LVEF were defined as primary endpoints. Key secondary endpoints included VO_2_ at AT and RCP, LVEDd, NT-proBNP, hs-troponin, CRP, selected body-composition outcomes, and 6MWT distance. The complete exploratory multidomain dataset, including additional CPET, echocardiographic, biochemical, body-composition, functional, and HRQoL variables, is provided in Supplementary Table S1. Values are presented as median (interquartile range). Within-group changes over time were assessed using the Friedman test. Post hoc pairwise comparisons were performed using Dunn’s test. Superscripts indicate significant differences compared with T1 (a), T2 (b), and T3 (c) (*p* < 0.05 after correction).

**Table 3:** Between-group comparisons of primary and key secondary outcomes across four time points (T1–T4).

Outcome	Time Point	U	Rank-Biserial r	95% CI for r		P (Holm-Adjusted)
				Lower	Upper	
Cardiopulmonary outcomes						
AT: VO_2_/kg (mL·kg^−1^·min^−1^)	T1	171.000	0.527	0.163	0.764	0.051
	T2	183.000	0.634	0.316	0.824	0.041
	T3	163.000	0.455	0.070	0.722	0.074
	T4	167.000	0.491	0.116	0.744	0.074
RCP: VO_2_/kg (mL·kg^−1^·min^−1^)	T1	172.000	0.536	0.175	0.769	0.060
	T2	166.500	0.487	0.110	0.741	0.111
	T3	157.000	0.402	0.005	0.689	0.112
	T4	152.000	0.357	−0.047	0.661	0.114
VO_2_peak: VO_2_/kg (mL·kg^−1^·min^−1^)	T1	152.500	0.362	−0.042	0.664	0.261
	T2	159.000	0.420	0.026	0.700	0.261
	T3	162.000	0.446	0.059	0.717	0.252
	T4	149.000	0.330	−0.078	0.644	0.281
Echocardiographic outcomes						
LVEDd (mm)	T1	73.500	−0.344	−0.652	0.063	0.336
	T2	79.500	−0.290	−0.617	0.122	0.336
	T3	77.000	−0.313	−0.632	0.097	0.336
	T4	56.000	−0.500	−0.749	−0.128	0.080
LVEF (%)	T1	220.500	0.969	0.929	0.986	<0.001
	T2	191.500	0.710	0.435	0.864	<0.001
	T3	169.500	0.513	0.145	0.757	0.012
	T4	138.500	0.237	−0.178	0.580	0.120
Biochemical outcomes						
NT-proBNP (pg·mL^−1^)	T1	31.000	−0.723	−0.871	−0.457	0.002
	T2	38.500	−0.656	−0.836	−0.350	0.004
	T3	48.500	−0.567	−0.787	−0.218	0.009
	T4	24.000	−0.786	−0.902	−0.564	0.001
CRP (mg·L^−1^)	T1	113.500	0.013	−0.386	0.409	1.000
	T2	115.000	0.027	−0.375	0.420	1.000
	T3	176.000	0.571	0.225	0.790	0.032
	T4	158.000	0.411	0.016	0.695	0.174
hs-troponin (ng·L^−1^)	T1	53.000	−0.527	−0.764	−0.163	0.015
	T2	20.000	−0.821	−0.919	−0.629	<0.001
	T3	26.500	−0.763	−0.891	−0.525	<0.001
	T4	11.000	−0.902	−0.956	−0.786	<0.001
Body composition outcomes						
Body weight (kg)	T1	111.000	−0.009	−0.405	0.390	1.000
	T2	114.500	0.022	−0.379	0.416	1.000
	T3	108.000	−0.036	−0.427	0.367	1.000
	T4	108.000	−0.036	−0.427	0.367	1.000
Body fat (%)	T1	134.500	0.201	−0.214	0.554	1.000
	T2	121.000	0.152	−0.267	0.524	1.000
	T3	126.500	0.129	−0.283	0.501	0.880
	T4	142.000	0.268	−0.145	0.602	1.000
Muscle mass (kg)	T1	110.000	−0.018	−0.413	0.383	1.000
	T2	115.000	0.027	−0.375	0.420	1.000
	T3	117.500	0.049	−0.356	0.438	1.000
	T4	115.500	0.031	−0.371	0.424	0.432
Functional outcomes						
6MWT (m)	T1	139.500	0.246	−0.169	0.586	0.908
	T2	140.000	0.250	−0.164	0.589	0.908
	T3	141.500	0.263	−0.150	0.598	0.908
	T4	140.500	0.254	−0.159	0.592	0.908

Between-group comparisons for primary and key secondary outcomes were performed using the Mann–Whitney U test at each time point. Effect size is reported as the rank-biserial correlation (r) with 95% confidence intervals. *p* values were adjusted for multiple comparisons using the Holm–Bonferroni method. The complete exploratory between-group comparison dataset is provided in Supplementary Table S2. Statistically significant differences are defined as Holm-adjusted *p* < 0.05.

## Data Availability

The data supporting the findings of this study are available from the corresponding author upon reasonable request, in accordance with the participants’ consent and data-sharing agreements.

## Supplementary Materials

The following supporting information can be downloaded at: https://www.mdpi.com/article/10.3390/jcm15124545/s1. Supplementary Table S1. Longitudinal changes in cardiopulmonary, echocardiographic, biochemical, body composition, functional, and health-related quality of life outcomes with effect sizes. Supplementary Table S2: Complete between-group comparisons across cardiopulmonary, echocardiographic, biochemical, body-composition, functional, and health-related quality of life outcomes at each assessment time point.

## Abbreviations

The following abbreviations are used in this manuscript:

6MWT	Six-Minute Walk Test
AT	Anaerobic Threshold (First Ventilatory Threshold)
BMI	Body Mass Index
CPET	Cardiopulmonary Exercise Test
CRP	C-reactive Protein
CTRL	Control Group
CVD	Cardiovascular Disease
ebCR	Exercise-Based Cardiac Rehabilitation
ECG	Electrocardiogram
LVEF	Left Ventricular Ejection Fraction
E/A	Ratio of Early (E) to Late (A) Ventricular Filling Velocity
E/e′	Ratio of Early Diastolic Filling Velocity to Mitral Annular Velocity
ESC	European Society of Cardiology
fT3	Free Triiodothyronine
fT4	Free Thyroxine
HDL	High-Density Lipoprotein
HIIT	High-Intensity Interval Training
HR	Heart Rate
HRpeak	Peak Heart Rate
HRQoL	Health-Related Quality of Life
hs-troponin	High-Sensitivity Cardiac Troponin
IVS	Interventricular Septum Thickness
LDL	Low-Density Lipoprotein
LV	Left Ventricle/Left Ventricular
LVEDd	Left Ventricular End-Diastolic Diameter
MI	Myocardial Infarction
MICT	Moderate-Intensity Continuous Training
NT-proBNP	N-terminal pro-B-type Natriuretic Peptide
PO	Power Output
PW	Posterior Wall Thickness
RAND-36	RAND 36-Item Health Survey
RCP	Respiratory Compensation Point (Second Ventilatory Threshold)
RPE	Rating of Perceived Exertion
STEMI	ST-Elevation Myocardial Infarction
TSH	Thyroid-Stimulating Hormone
VE	Minute Ventilation
VCO_2_	Carbon Dioxide Output
VO_2_	Oxygen Uptake
VO_2_peak	Peak Oxygen Uptake
VO_2_/HR	Oxygen Pulse
VO_2_/WR	Oxygen Uptake to Work Rate Slope
WHR	Waist-to-Hip Ratio
