# Supplementary material for: Time-Course of Physiological Adaptations to High-Intensity Interval Training-Based Cardiac Rehabilitation After Myocardial Infarction
Source: J Clin Med. 2026 Jun 11;15(12):4545. doi: 10.3390/jcm15124545 (PMC13301535; doi:10.3390/jcm15124545)
Supplement: Supplementary file 1 [file jcm-15-04545-s001.zip › jcm-4349441-supplementary.pdf]

**Supplementary Table S2:** Complete between-group comparisons across cardiopulmonary, echocardiographic, biochemical, body-composition, functional, and health-related quality-of-life outcomes at each assessment time point.

| Outcome | Time point | U | Rank-biserial r | 95% CI for r Lower | Upper | P (holm-adjusted) |
|---|---|---|---|---|---|---|
| **Cardiopulmonary outcomes** | | | | | | |
| AT: $VO_2$/kg (mL·kg⁻¹·min⁻¹) | T1 | 171.000 | 0.527 | 0.163 | 0.764 | 0.051 |
| | T2 | 183.000 | 0.634 | 0.316 | 0.824 | 0.041 |
| | T3 | 163.000 | 0.455 | 0.070 | 0.722 | 0.074 |
| | T4 | 167.000 | 0.491 | 0.116 | 0.744 | 0.074 |
| AT: HR (bpm) | T1 | 221.000 | 0.973 | 0.939 | 0.988 | <0.001 |
| | T2 | 175.500 | 0.567 | 0.218 | 0.787 | 0.009 |
| | T3 | 187.500 | 0.674 | 0.378 | 0.845 | <0.001 |
| | T4 | 202.000 | 0.804 | 0.596 | 0.910 | <0.001 |
| RCP: $VO_2$/kg (mL·kg⁻¹·min⁻¹) | T1 | 172.000 | 0.536 | 0.175 | 0.769 | 0.060 |
| | T2 | 166.500 | 0.487 | 0.110 | 0.741 | 0.111 |
| | T3 | 157.000 | 0.402 | 0.005 | 0.689 | 0.112 |
| | T4 | 152.000 | 0.357 | -0.047 | 0.661 | 0.114 |
| RCP: HR (bpm) | T1 | 223.000 | 0.991 | 0.979 | 0.996 | <0.001 |
| | T2 | 197.000 | 0.759 | 0.517 | 0.888 | <0.001 |
| | T3 | 194.500 | 0.737 | 0.479 | 0.877 | <0.001 |
| | T4 | 194.500 | 0.737 | 0.479 | 0.877 | <0.001 |
| $VO_{2peak}$: $VO_2$/kg (mL·kg⁻¹·min⁻¹) | T1 | 152.500 | 0.362 | -0.042 | 0.664 | 0.261 |
| | T2 | 159.000 | 0.420 | 0.026 | 0.700 | 0.261 |
| | T3 | 162.000 | 0.446 | 0.059 | 0.717 | 0.252 |
| | T4 | 149.000 | 0.330 | -0.078 | 0.644 | 0.281 |
| $VO_{2peak}$: HR(bpm) | T1 | 216.000 | 0.929 | 0.842 | 0.969 | <0.001 |
| | T2 | 206.000 | 0.839 | 0.663 | 0.927 | <0.001 |
| | T3 | 205.500 | 0.835 | 0.654 | 0.925 | <0.001 |
| | T4 | 195.500 | 0.746 | 0.494 | 0.882 | <0.001 |
| $VO_{2peak}$: Power (W) | T1 | 152.000 | 0.357 | -0.047 | 0.661 | 0.126 |
| | T2 | 161.500 | 0.442 | 0.054 | 0.714 | 0.072 |
| | T3 | 153.000 | 0.366 | -0.037 | 0.667 | 0.108 |
| | T4 | 136.000 | 0.214 | -0.201 | 0.564 | 0.255 |
| VE/$VCO_2$ slope | T1 | 126.000 | 0.125 | -0.287 | 0.498 | 0.680 |
| | T2 | 68.000 | -0.393 | -0.684 | 0.006 | 0.348 |
| | T3 | 107.500 | -0.040 | -0.431 | 0.363 | 0.705 |
| | T4 | 73.000 | -0.348 | -0.655 | 0.057 | 0.612 |
| $VO_2$/WR (mL·min⁻¹·W⁻¹) | T1 | 145.000 | 0.295 | -0.117 | 0.620 | 0.441 |
| | T2 | 152.000 | 0.357 | -0.047 | 0.661 | 0.340 |
| | T3 | 133.000 | 0.188 | -0.227 | 0.545 | 1.000 |
| | T4 | 107.000 | -0.045 | -0.435 | 0.359 | 1.000 |
| $VO_2$/HR (mL·beat⁻¹) | T1 | 104.500 | -0.067 | -0.453 | 0.340 | 1.000 |
| | T2 | 112.500 | 0.004 | -0.394 | 0.401 | 1.000 |
| | T3 | 121.500 | 0.085 | -0.324 | 0.467 | 1.000 |
| | T4 | 92.500 | -0.174 | -0.535 | 0.240 | 1.000 |

| | | | | | | |
|---|---|---|---|---|---|---|
| **Echocardiographic outcomes** | | | | | | |
| LVEDd (mm) | T1 | 73.500 | -0.344 | -0.652 | 0.063 | 0.336 |
| | T2 | 79.500 | -0.290 | -0.617 | 0.122 | 0.336 |
| | T3 | 77.000 | -0.313 | -0.632 | 0.097 | 0.336 |
| | T4 | 56.000 | -0.500 | -0.749 | -0.128 | 0.080 |
| LVEF (%) | T1 | 220.500 | 0.969 | 0.929 | 0.986 | <0.001 |
| | T2 | 191.500 | 0.710 | 0.435 | 0.864 | <0.001 |
| | T3 | 169.500 | 0.513 | 0.145 | 0.757 | 0.012 |
| | T4 | 138.500 | 0.237 | -0.178 | 0.580 | 0.120 |
| IVS (mm) | T1 | 88.000 | -0.214 | -0.564 | 0.201 | 0.952 |
| | T2 | 87.000 | -0.223 | -0.570 | 0.192 | 0.952 |
| | T3 | 105.000 | -0.063 | -0.449 | 0.344 | 0.952 |
| | T4 | 86.500 | -0.228 | -0.573 | 0.187 | 0.952 |
| PW(mm) | T1 | 104.000 | -0.071 | -0.456 | 0.336 | 1.000 |
| | T2 | 75.500 | -0.326 | -0.641 | 0.083 | 0.456 |
| | T3 | 106.500 | -0.049 | -0.438 | 0.356 | 1.000 |
| | T4 | 96.000 | -0.143 | -0.512 | 0.270 | 1.000 |
| E/A ratio | T1 | 86.500 | -0.228 | -0.573 | 0.187 | 0.735 |
| | T2 | 71.500 | -0.362 | -0.664 | 0.042 | 0.376 |
| | T3 | 84.000 | -0.250 | -0.589 | 0.164 | 0.735 |
| | T4 | 107.000 | -0.045 | -0.435 | 0.359 | 0.846 |
| E/e’ | T1 | 41.000 | -0.634 | -0.824 | -0.316 | 0.012 |
| | T2 | 47.000 | -0.580 | -0.795 | -0.237 | 0.021 |
| | T3 | 79.000 | -0.295 | -0.620 | 0.117 | 0.344 |
| | T4 | 90.500 | -0.192 | -0.548 | 0.223 | 0.380 |
| **Biochemical outcomes** | | | | | | |
| Triglycerides (mmol·L⁻¹) | T1 | 100.500 | -0.103 | -0.481 | 0.308 | 1.000 |
| | T2 | 128.500 | 0.147 | -0.266 | 0.515 | 1.000 |
| | T3 | 123.000 | 0.098 | -0.312 | 0.477 | 1.000 |
| | T4 | 121.500 | 0.085 | -0.324 | 0.467 | 1.000 |
| Cholesterol (mmol·L⁻¹) | T1 | 195.000 | 0.741 | 0.487 | 0.880 | 0.002 |
| | T2 | 196.500 | 0.754 | 0.510 | 0.886 | 0.002 |
| | T3 | 180.500 | 0.612 | 0.283 | 0.812 | 0.006 |
| | T4 | 163.500 | 0.668 | 0.356 | 0.846 | 0.006 |
| HDL (mmol·L⁻¹) | T1 | 136.500 | 0.219 | 0.356 | 0.846 | 0.945 |
| | T2 | 151.000 | 0.348 | -0.196 | 0.567 | 0.428 |
| | T3 | 132.500 | 0.183 | -0.057 | 0.655 | 0.945 |
| | T4 | 117.500 | 0.049 | -0.232 | 0.541 | 0.945 |
| LDL (mmol·L⁻¹) | T1 | 199.000 | 0.777 | 0.549 | 0.897 | <0.001 |
| | T2 | 201.000 | 0.795 | 0.580 | 0.906 | <0.001 |
| | T3 | 192.000 | 0.714 | 0.442 | 0.866 | 0.002 |
| | T4 | 190.000 | 0.696 | 0.413 | 0.857 | 0.002 |
| LDL/HDL | T1 | 198.000 | 0.768 | 0.533 | 0.893 | 0.001 |
| | T2 | 192.000 | 0.714 | 0.442 | 0.866 | 0.002 |
| | T3 | 189.500 | 0.692 | 0.406 | 0.855 | 0.002 |
| | T4 | 192.000 | 0.714 | 0.442 | 0.866 | 0.002 |
| | T1 | 31.000 | -0.723 | -0.871 | -0.457 | 0.002 |

| | | | | | | |
|---|---|---|---|---|---|---|
| NT-proBNP (pg·mL⁻¹) | T2 | 38.500 | -0.656 | -0.836 | -0.350 | 0.004 |
| | T3 | 48.500 | -0.567 | -0.787 | -0.218 | 0.009 |
| | T4 | 24.000 | -0.786 | -0.902 | -0.564 | 0.001 |
| CRP (mg·L⁻¹) | T1 | 113.500 | 0.013 | -0.386 | 0.409 | 1.000 |
| | T2 | 115.000 | 0.027 | -0.375 | 0.420 | 1.000 |
| | T3 | 176.000 | 0.571 | 0.225 | 0.790 | 0.032 |
| | T4 | 158.000 | 0.411 | 0.016 | 0.695 | 0.174 |
| hs-troponin (ng·L⁻¹) | T1 | 53.000 | -0.527 | -0.764 | -0.163 | 0.015 |
| | T2 | 20.000 | -0.821 | -0.919 | -0.629 | <0.001 |
| | T3 | 26.500 | -0.763 | -0.891 | -0.525 | <0.001 |
| | T4 | 11.000 | -0.902 | -0.956 | -0.786 | <0.001 |
| TSH (mIU·L⁻¹) | T1 | 169.000 | 0.509 | 0.139 | 0.754 | 0.052 |
| | T2 | 160.500 | 0.433 | 0.043 | 0.709 | 0.054 |
| | T3 | 172.500 | 0.540 | 0.181 | 0.772 | 0.052 |
| | T4 | 165.500 | 0.478 | 0.099 | 0.736 | 0.054 |
| fT3(pmol·L⁻¹) | T1 | 60.500 | -0.460 | -0.725 | -0.076 | 0.102 |
| | T2 | 101.500 | -0.094 | -0.474 | 0.316 | 1.000 |
| | T3 | 122.500 | 0.094 | -0.316 | 0.474 | 1.000 |
| | T4 | 47.000 | -0.580 | -0.795 | -0.237 | 0.028 |
| fT4 (pmol·L⁻¹) | T1 | 61.500 | -0.451 | -0.720 | -0.065 | 0.148 |
| | T2 | 72.000 | -0.357 | -0.661 | 0.047 | 0.300 |
| | T3 | 91.500 | -0.183 | -0.541 | 0.232 | 0.810 |
| | T4 | 117.000 | 0.045 | -0.359 | 0.435 | 0.851 |
| eGFR (mL·min⁻¹·1.73m²⁻¹) | T1 | 153.500 | 0.371 | -0.032 | 0.670 | 0.352 |
| | T2 | 132.000 | 0.179 | -0.236 | 0.538 | 0.834 |
| | T3 | 140.500 | 0.254 | -0.159 | 0.592 | 0.729 |
| | T4 | 106.000 | -0.054 | -0.442 | 0.352 | 0.834 |

**Body composition outcomes**

| | | | | | | |
|---|---|---|---|---|---|---|
| Body mass (kg) | T1 | 111.000 | -0.009 | -0.405 | 0.390 | 1.000 |
| | T2 | 114.500 | 0.022 | -0.379 | 0.416 | 1.000 |
| | T3 | 108.000 | -0.036 | -0.427 | 0.367 | 1.000 |
| | T4 | 108.000 | -0.036 | -0.427 | 0.367 | 1.000 |
| Body fat (%) | T1 | 134.500 | 0.201 | -0.214 | 0.554 | 1.000 |
| | T2 | 121.000 | 0.152 | -0.267 | 0.524 | 1.000 |
| | T3 | 126.500 | 0.129 | -0.283 | 0.501 | 0.880 |
| | T4 | 142.000 | 0.268 | -0.145 | 0.602 | 1.000 |
| Muscle mass (kg) | T1 | 110.000 | -0.018 | -0.413 | 0.383 | 1.000 |
| | T2 | 115.000 | 0.027 | -0.375 | 0.420 | 1.000 |
| | T3 | 117.500 | 0.049 | -0.356 | 0.438 | 1.000 |
| | T4 | 115.500 | 0.031 | -0.371 | 0.424 | 0.432 |
| Visceral fat (index) | T1 | 79.500 | -0.290 | -0.617 | 0.122 | 0.432 |
| | T2 | 80.000 | -0.286 | -0.614 | 0.126 | 0.140 |
| | T3 | 61.000 | -0.455 | -0.722 | -0.070 | 0.432 |
| | T4 | 76.500 | -0.317 | -0.635 | 0.092 | 1.000 |

| | | | | | | |
|---|---|---|---|---|---|---|
| **Functional outcomes** | | | | | | |
| 6MWT (m) | T1 | 139.500 | 0.246 | -0.169 | 0.586 | 0.908 |
| | T2 | 140.000 | 0.250 | -0.164 | 0.589 | 0.908 |
| | T3 | 141.500 | 0.263 | -0.150 | 0.598 | 0.908 |
| | T4 | 140.500 | 0.254 | -0.159 | 0.592 | 0.908 |
| **Health related quality of life** | | | | | | |
| Physical functioning | T1 | 132.500 | 0.183 | -0.232 | 0.541 | 0.403 |
| | T2 | 170.500 | 0.522 | 0.157 | 0.762 | 0.028 |
| | T3 | 198.500 | 0.772 | 0.541 | 0.895 | 0.001 |
| | T4 | 182.500 | 0.629 | 0.309 | 0.822 | 0.009 |
| RL: physical health | T1 | 165.500 | 0.478 | 0.099 | 0.736 | 0.064 |
| | T2 | 144.000 | 0.286 | -0.126 | 0.614 | 0.300 |
| | T3 | 144.500 | 0.290 | -0.122 | 0.617 | 0.300 |
| | T4 | 122.500 | 0.000 | -0.398 | 0.398 | 0.619 |
| RL: Emotional problems | T1 | 134.500 | 0.201 | -0.214 | 0.554 | 1.000 |
| | T2 | 114.500 | 0.022 | -0.379 | 0.416 | 1.000 |
| | T3 | 121.500 | 0.000 | -0.398 | 0.398 | 1.000 |
| | T4 | 109.000 | -0.027 | -0.420 | 0.375 | 1.000 |
| Energy/fatigue | T1 | 126.500 | 0.129 | -0.283 | 0.501 | 0.964 |
| | T2 | 133.000 | 0.188 | -0.227 | 0.545 | 1.000 |
| | T3 | 137.000 | 0.223 | -0.192 | 0.570 | 1.000 |
| | T4 | 140.500 | 0.254 | -0.159 | 0.592 | 1.000 |
| Emotional well-being | T1 | 128.500 | 0.147 | -0.266 | 0.515 | 0.758 |
| | T2 | 133.500 | 0.192 | -0.223 | 0.548 | 0.758 |
| | T3 | 157.500 | 0.406 | 0.010 | 0.692 | 0.236 |
| | T4 | 151.000 | 0.348 | -0.057 | 0.655 | 0.324 |
| Social functioning | T1 | 181.000 | 0.616 | 0.289 | 0.814 | 0.012 |
| | T2 | 152.000 | 0.357 | -0.047 | 0.661 | 0.252 |
| | T3 | 136.000 | 0.214 | -0.201 | 0.564 | 0.608 |
| | T4 | 129.500 | 0.156 | -0.257 | 0.522 | 0.608 |
| Pain | T1 | 154.000 | 0.375 | -0.027 | 0.672 | 0.150 |
| | T2 | 151.000 | 0.348 | -0.057 | 0.655 | 0.457 |
| | T3 | 166.000 | 0.482 | 0.104 | 0.738 | 0.076 |
| | T4 | 162.500 | 0.451 | 0.065 | 0.720 | 0.076 |
| General health | T1 | 171.500 | 0.531 | 0.169 | 0.767 | 0.026 |
| | T2 | 163.500 | 0.460 | 0.076 | 0.725 | 0.032 |
| | T3 | 181.000 | 0.616 | 0.289 | 0.814 | 0.016 |
| | T4 | 178.000 | 0.589 | 0.250 | 0.800 | 0.018 |

Between-group comparisons were performed using the Mann–Whitney U test at each assessment time point (T1–T4). Effect size is reported as the rank-biserial correlation (r) with 95% confidence intervals. P values were adjusted for multiple comparisons using the Holm-Bonferroni method. Outcomes not included in the main primary/key secondary endpoint table are reported for exploratory and hypothesis-generating purposes. Statistically significant differences were defined as Holm-adjusted $p < 0.05$.
