# Supplementary material for: Time-Course of Physiological Adaptations to High-Intensity Interval Training-Based Cardiac Rehabilitation After Myocardial Infarction
Source: J Clin Med. 2026 Jun 11;15(12):4545. doi: 10.3390/jcm15124545 (PMC13301535; doi:10.3390/jcm15124545)
Supplement: Supplementary file 1 [file jcm-15-04545-s001.zip › jcm-4349441-supplementary (1).pdf]

Supplementary Table 1. Longitudinal changes in cardiopulmonary, echocardiographic, biochemical, body composition, functional, and health-related quality of life outcomes

	Group	T1	T2	T3	T4	Friedman χ²(3)	p (Friedman)	*Post-hoc* Dunn	Kendall's W
**Cardiopulmonary outcomes**									
AT: VO2 (mL·kg⁻¹·min⁻¹)	MI (n = 16)	12.1 (10.7-13.6)	13.4 (12.4-15.5)	14.2 (12.2-17.8)	17.5 (15.5-20.5)	39.72	<0.001	T1 vs. T3: p = 0.0006<br>T1 vs. T4: p <0.0001<br>T2 vs. T4: p = 0.0006	0.83
	CTRL (n = 14)	15.9 (13.3-19.2)	17.8 (14.9-19.6)	19.5 (17.6-20.9)	22.1 (21.3-25.4)	23.4	<0.001	T1 vs. T4: p <0.0001<br>T2 vs. T4: p = 0.0046	0.56
AT: HR (bpm)	MI (n = 16)	89 (84-97)	101 (88-116)	101 (88-116)	106 (94-109)	24	<0.001	T1 vs. T2: p = 0.0061<br>T1 vs. T3: p = 0.0061<br>T1 vs. T4: p <0.0001	0.50
	CTRL (n = 14)	126 (111-138)	121 (106-130)	122 (120-134)	133 (118-143)	7.356	0.061	/	0.18
RCP: VO2 (mL·kg⁻¹·min⁻¹)	MI (n = 16)	14.6 (12.3-17.1)	18.2 (14.2-21.2)	18.4 (15.0-25.4)	20.5 (19.5-27.8)	40.81	<0.001	T1 vs. T3: p = 0.0003<br>T1 vs. T4: p <0.0001<br>T2 vs. T4: p = 0.0006	0.85
	CTRL (n = 14)	19.3 (15.9-23.6)	21.6 (19.3-25.2)	24.1 (21.9-26.8)	25.5 (23.8-28.5)	28.89	<0.001	T1 vs. T3: p = 0.0077<br>T1 vs. T4: p ≤0.0001<br>T2 vs. T4: p = 0.0027	0.69
RCP: HR (bpm)	MI (n = 16)	107 (99-112)	116 (103-128)	116 (105-130)	124 (109-128)	18.69	<0.001	T1 vs. T3: p = 0.0037<br>T1 vs. T4: p = 0.0008	0.39
	CTRL (n = 14)	142 (138-159)	147 (134-151)	148 (144-152)	148 (143-157)	2.511	0.473	/	0.06
VO2peak: VO2 (mL·kg⁻¹·min⁻¹)	MI (n = 16)	20.1 (15.6-22.7)	21.2 (16.9-25.1)	22.2 (19.5-25.3)	24.5 (22.7-32.3)	39.6	<0.001	T1 vs. T3: p = 0.0061<br>T1 vs. T4: p <0.0001<br>T2 vs. T4: p <0.0001<br>T3 vs. T4: p = 0.0370	0.83
	CTRL (n = 14)	22.3 (19.5-27.1)	24.3 (22.8-29.4)	26.8 (23.1-28.1)	27.6 (25.3-31.8)	27.69	<0.001	T1 vs. T4: p <0.0001<br>T2 vs. T4: p = 0.0005<br>T3 vs. T4: p = 0.0325	0.66
VO2peak: HR (bpm)	MI (n = 16)	126 (119-137)	131 (117-143)	132 (122-141)	144 (133-151)	9.755	0.021	T1 vs. T4: p = 0.0155	0.20
	CTRL (n = 14)	164 (158-180)	167 (155-170)	170 (157-177)	170 (157-178)	2.2	0.532	/	0.05
VO2peak: Power	MI (n = 16)	140 (119-170)	157 (131-186)	166 (145-208)	196 (156-218)	45.9	<0.001	T1 vs. T3: p = 0.0002<br>T1 vs. T4: p <0.0001<br>T2 vs. T4: p <0.0001	0.96

(W)	CTRL (n = 14)	180 (122-212)	190 (146-235)	207 (165-239)	217 (146-253)	32.78	<0.001	T1 vs. T3: p = 0.0005<br>T1 vs. T4: p < 0.0001<br>T2 vs. T4: p = 0.0127	0.78
VE/VCO2 slope	MI (n = 16)	26.8 (25.2-28.1)	27.7 (26.2-29.2)	29.2 (25.2-30.2)	29.8 (27.9-30.4)	10.13	0.018	T1 vs. T4: p = 0.0124	0.21
	CTRL (n = 14)	27.1 (25.2-29.6)	25.9 (24.2-27.4)	28.5 (27.5-29.5)	28.2 (26.6-29.2)	7.891	0.048	T2 vs. T3: p = 0.0406	0.19
VO2/WR slope ($mL\cdot min^{-1}\cdot W^{-1}$)	MI (n = 16)	8.8 (7.1-9.8)	7.9 (7.4-9.2)	8.8 (8.0-9.4)	9.3 (8.8-9.8)	10.73	0.013	T2 vs. T4: p = 0.0098	0.22
	CTRL (n = 14)	9.3 (8.6-10.1)	8.9 (8.2-9.8)	9.2 (8.2-9.5)	9.3 (8.8-9.8)	4.029	0.258	/	0.10
VO_2/HR ($mL\cdot beat^{-1}$)	MI (n = 16)	13.2 (9.2-14.3)	13.8 (11.8-16.9)	14.8 (12.7-16.7)	15.2 (10.9-18.2)	18.48	<0.001	T1 vs. T4: p = 0.0002<br>T2 vs. T4: p = 0.0325	0.39
	CTRL (n = 14)	13.1 (11.4-15.7)	14.2 (12.5-15.7)	14.3 (13.3-17.2)	16.9 (14.7-18.4)	20.43	<0.001	T1 vs. T4: p = 0.0006<br>T2 vs. T4: p = 0.0013	0.49
**Echocardiographic outcomes**									
LVEDd (mm)	MI (n = 16)	52.0 (47.3–55.0)	51.5 (48.3–55.0)	51.0 (48.0–54.5)	53.0 (50.5–55.0)	4.70	0.195	/	0.10
	CTRL (n = 14)	48.0 (46.3–50.8)	48.5 (47.3–51.5)	49.0 (44.5–51.0)	50.0 (47.5–51.3)	17.35	<0.001	T1 vs. T4: p = 0-0046	0.41
LVEF (%)	MI (n = 16)	52.5 (50.0–55.0)	57.5 (55.2–58.7)	58.0 (57.2–60.0)	60.0 (55.8–60.0)	36.52	<0.001	T1 vs. T2: p = 0.0242<br>T1 vs. T3: p <0.0001<br>T1 vs. T4: p <0.0001	0.76
	CTRL (n = 14)	60.0 (55.7–65.5)	61.0 (57.0–66.3)	62.0 (58.0–63.7)	62.0 (59.5–67.7)	4.6	0.2	/	0.11
IVS (mm)	MI (n = 16)	11.0 (10.0–11.7)	11.0 (10.0–11.8)	11.0 (10.0–12.0)	11.0 (10.0–12.0)	8.486	0.037	No significant pairwise comparisons	0.18
	CTRL (n = 14)	10.5 (9.8–11.0)	10.5 (9.8–11.0)	11.0 (10.0–11.0)	11.0 (10.8–11.0)	10.8	0.013	No significant pairwise comparisons	0.26
PW (mm)	MI (n = 16)	10.0 (9.3–11.0)	11.0 (10.0–11.0)	11.0 (10.0–11.0)	11.0 (10.0–11.0)	5.188	0.159	/	0.11

PW (mm)	CTRL (n = 14)	10.0 (9.7–10.3)	10.0 (9.7–10.3)	10.0 (10.0–11.0)	10.0 (10.0–11.0)	5.143	0.161	/	0.12
E/A ratio	MI (n = 16)	0.89 (0.78–0.98)	0.90 (0.82–0.98)	0.90 (0.83–0.98)	0.90 (0.84–0.98)	5.204	0.157	/	0.11
	CTRL (n = 14)	0.84 (0.80–0.88)	0.84 (0.80–0.89)	0.88 (0.87–0.89)	0.90 (0.88–0.96)	23.23	<0.001	T1 vs. T4: p = 0.0027<br>T2 vs. T4: p = 0.0027	0.55
E/e'	MI (n = 16)	8.74 (8.2–9.1)	8.75 (7.7–9.2)	8.1 (7.0–9.2)	7.78 (7.3–9.2)	8.303	0.04	No significant pairwise comparisons	0.17
	CTRL (n = 14)	6.66 (6.3–7.1)	6.66 (6.3–7.1)	7.15 (7.1–7.6)	7.73 (6.9–7.7)	5.269	0.153	/	0.13
**Biochemical outcomes**									
Triglycerides (mmol·L⁻¹)	MI (n = 16)	1.5 (0.9-2.4)	1.1 (0.8-1.5)	1.1 (0.8-1.8)	1.3 (0.8-1.6)	8.66	0.034	No significant pairwise comparisons	0.18
	CTRL (n = 14)	1.4 (0.8-1.8)	1.3 (0.8-1.7)	1.3 (1.1-1.6)	1.4 (1.1-1.6)	1.279	0.737	/	0.03
HDL (mmol·L⁻¹)	MI (n = 16)	1.2 (1.1-1.5)	1.3 (1.0-1.5)	1.4 (1.2-1.6)	1.4 (1.1-1.5)	6.07	0.108	/	0.13
	CTRL (n = 14)	1.5 (1.0-1.9)	1.6 (1.1-1.9)	1.4 (1.3-1.7)	1.3 (1.2-1.7)	2.4217	0.49	/	0.06
HDL/CHOL	MI (n = 16)	0.4 (0.3-0.4)	0.4 (0.3-0.4)	0.4 (0.3-0.4)	0.4 (0.3-0.4)	4.80	0.187	/	0.10
	CTRL (n = 14)	0.3 (0.2-0.3)	0.3 (0.2-0.3)	0.3 (0.3-0.3)	0.3 (0.3–-0.3)	8.833	0.032	No significant pairwise comparisons	0.21
LDL (mmol·L⁻¹)	MI (n = 16)	1.7 (1.3-2.3)	1.7 (1.4-2.1)	1.8 (1.3-2.6)	1.7 (1.5-1.9)	2.559	0.465	/	0.05
	CTRL (n = 14)	3.5 (2.8-4.4)	3.4 (2.8-4.1)	3.0 (2.3-3.5)	3.0 (2.6-3.6)	14.65	0.002	T1 vs. T4: p = 0.0077	0.35
LDL/HDL	MI (n = 16)	1.3 (1.1-1.8)	1.3 (1.2-1.7)	1.3 (1.1-1.6)	1.3 (1.2-1.6)	5.646	0.13	/	0.12

LDL/HDL	CTRL (n = 14)	2.4 (1.9-2.9)	2.2 (1.8-2.6)	2.0 (1.8-2.4)	2.1 (1.8-2.4)	10.49	0.015	T1 vs. T3: p = 0.0259	0.25
NT-proBNP (pg·mL⁻¹)	MI (n = 16)	90.5 (81.2-227.3)	139.5 (64.8-263.8)	130.0 (53.3-282.5)	115.0 (68.5-206.8)	2.312	0.51	/	0.05
	CTRL (n = 14)	44.5 (20.8-50.5)	41.0 (25.0-73.8)	55.0 (29.8-64.8)	39.5 (21.0-63.5)	2.289	0.515	/	0.05
CRP (mg·L⁻¹)	MI (n = 16)	2.1 (0.4-3.1)	1.3 (0.3-3.3)	1.0 (0.4-1.7)	0.7 (0.2-1.7)	11.80	0.008	T1 vs. T4: p = 0.0194	0.25
	CTRL (n = 14)	1.2 (0.6-3.9)	1.0 (0.7-2.3)	2.9 (1.1-3.3)	1.2 (1.0-2.3)	6.088	0.107	/	0.14
hs-troponin (ng·L⁻¹)	MI (n = 16)	8.0 (6.3-9.4)	8.9 (7.7-11.5)	10.2 (8.6-11.4)	10.5 (8.4-12.6)	25.38	<0.001	T1 vs. T2: p = 0.0300<br>T1 vs. T3: p <0.0001<br>T1 vs. T4: p <0.0001	0.53
	CTRL (n = 14)	5.0 (4.7-7.3)	5.8 (5.4-6.9)	5.0 (4.8-6.9)	4.9 (4.3-6.8)	11.23	0.011	T2 vs. T4: p = 0.0127	0.27
TSH (mIU·L⁻¹)	MI (n = 16)	1.0 (0.9-2.2)	1.4 (1.0-2.5)	1.3 (0.9-2.1)	1.3 (1.0-1.9)	3.778	0.286	/	0.08
	CTRL (n = 14)	2.2 (1.6-3.7)	1.9 (1.6-2.9)	2.5 (1.7-3.0)	1.8 (1.4-2.9)	4.286	0.232	/	0.10
fT3 (pmol·L⁻¹)	MI (n = 16)	5.2 (4.5-5.9)	5.0 (4.4-5.4)	4.6 (4.5-5.5)	5.1 (4.7-5.4)	0.98	0.806	/	0.02
	CTRL (n = 14)	4.5 (4.3—-4.7)	5.0 (4.5—-5.2)	4.8 (4.7—-5.1)	4.5 (4.1—-4.6)	15.93	0.001	T2 vs. T4: p = 0.0162<br>T3 vs. T4: p = 0.0099	0.38
fT4 (pmol·L⁻¹)	MI (n = 16)	16.2 (14.3-18.2)	15.2 (14.8-17.1)	15.4 (14.3-16.6)	13.9 (13.7-16.5)	13.34	0.004	T1 vs. T4: p = 0.0037	0.28
	CTRL (n = 14)	14.4 (13.2-15.1)	14.6 (12.7-15.4)	15.2 (13.6-15.8)	14.7 (13.6-15.6)	2.619	0.454	/	0.06
eGRF (mL·min⁻¹·1.73m2⁻¹)	MI (n = 16)	88.0 (64.8-93.5)	85.0 (67.8-90.8)	79.0 (73.0-87.3)	90.5 (76.5-93.8)	11.03	0.011	T3 vs. T4: p = 0.0155	0.23
	CTRL (n = 14)	93.0 (87.8-102.0)	87.0 (81.8-93.5)	84.0 (79.8-89.8)	89.0 (84.0-95.3)	13.12	0.004	T1 vs. T3: p = 0.0059	0.31

**Body composition outcomes**									
Body weight (kg)	MI (n = 16)	85.5 (79.0-89.9)	85.3 (78.2–88.8)	84.7 (77.7–90.3)	82.8 (76.9–90.5)	4.95	0.176	/	0.10
	CTRL (n = 14)	87.8 (66.9–103.2)	87.7 (67.5–102.9)	86.5 (66.2–100.5)	85.9 (66.1–99.2)	15.69	0.001	T1 vs. T3: p = 0.0385, T1 vs. T4: p= 0.0252	0.37
Body fat (%)	MI (n = 16)	25.7 (24.1-30.8)	25.70 (23.5-30.7)	25.80 (24.4-29.3)	24.80 (23.4-29.1)	18.27	<0.001	T1 vs. T4: p=0.0002	0.38
	CTRL (n = 14)	29.1 (22.6-33.5)	28.40 (22.2-33.4)	28.50 (20.8-31.8)	27.8 (22.5-32.8)	20.74	<0.001	T1 vs. T3: p = 0.0020<br>T1 vs. T4: p = 0.0011	0.49
Muscle mass (kg)	MI (n = 16)	61.3 (58.6-64.1)	61.4 (58.5-63.8)	61.8 (59.4-64.2)	61.1 (58.3-64.3)	0.375	0.945	/	0.01
	CTRL (n = 14)	61.2 (44.2-68.9)	62.5 (44.3-69.4)	62.8 (45.6-68.7)	62.6 (45.1-68.2)	6.8	0.079	/	0.16
Visceral Fat (index)	MI (n = 16)	10.5 (8.3-12.8)	10.5 (7.3-12.0)	11.0 (9.0-13.0)	10.0 (7.3-12.8)	13.08	0.005	No significant pairwise comparisons	0.27
	CTRL (n = 14)	7.5 (6.0-12.0)	7.5 (6.0-11.3)	7.5 (5.8-11.0)	7.5 (5.8-10.3)	7.4	0.06	/	0.18
**Functional outcomes**									
6 MWT (m)	MI (n = 16)	675 (607.3-746.3)	721 (638.8-768.8)	712.5 (661.8-798.8)	745.0 (662.5-811.3)	32.81	<0.001	T1 vs. T2: p = 0.0155<br>T1 vs. T3: p = 0.0002<br>T1 vs. T4: p <0.0001	0.68
	CTRL (n = 14)	714 (630.0-800.0)	730.5 (665.0-827.8)	762.5 (666.3-828.8)	775.0 (688.8-861.8)	18.7	<0.001	T1 vs. T4: p = 0.0001	0.45
**Health related quality of life**									
Physical functioning	MI (n = 16)	75.0 (57.5-88.8)	80.0 (71.3-93.7)	82.5 (56.3-93.8)	90.0 (80.0-95.0)	5.277	0.153		0.11
	CTRL (n = 14)	85.0 (51.5-100.0)	97.5 (90.0-100.0)	100 (95.0-100.0)	100 (95.0-100.0)	9.621	0.022	/	0.23

RL: physical health	MI (n = 16)	62.5 (25.0-100.0)	100.0 (50.0-100.0)	100.0 (75.0-100.0)	100.0 (56.3-100.0)	5.206	0.157	/	0.11
	CTRL (n = 14)	100 (75.6-100.0)	100 (93.7-100.0)	100 (100.0-100.0)	100 (75.0-100.0)	1.667	0.644	/	0.04
RL: emotional problems	MI (n = 16)	83.3 (8.3-100.0)	100 (41.7-100.0)	100.0 (75.0-100.0)	100.0 (100-100.0)	6.621	0.085	/	0.14
	CTRL (n = 14)	100 (58.3-100.0)	100 (58.3-100.0)	100 (100.0-100.0)	100 (91.67-100.0)	0.737	0.629	/	0.02
Energy/fatigue	MI (n = 16)	65.5 (50.0-70.0)	62.5 (48.8-75.0)	67.5 (43.7-73.8)	65.0 (52.5-75.0)	0.438	0.932	/	0.01
	CTRL (n = 14)	62.5 (53.7-76.3)	72.5 (53.8-81.3)	67.5 (58.8-86.3)	70.0 (60.0-85.0)	4.529	0.209	/	0.11
Emotional well-being	MI (n = 16)	70.0 (53.0-79.0)	70.0 (62.0-76.0)	68.0 (55.0-79.0)	72.0 (61.0-85.0)	0.438	0.932	/	0.01
	CTRL (n = 14)	78.0 (58.0-84.0)	84.0 (61.0-88.0)	86.0 (67.0-89.0)	84.0 (68.0-92.0)	9.676	0.022	/	0.23
Social functioning	MI (n = 16)	75.0 (62.5-87.5)	75.0 (75.9-96.9)	81.3 (65.6-100.0)	87.5 (62.5-100.0)	6.433	0.092	/	0.13
	CTRL (n = 14)	100.0 (84.4-100.0)	100.0 (84.4-100.0)	93.8 (75.0-100.0)	93.8 (75.0-100.0)	1.680	0.641	/	0.04
Pain	MI (n = 16)	77.5 (67.5-97.5)	90.0 (78.1-97.5)	83.8 (70.0-97.5)	78.5 (77.5-97.5)	5.000	0.172	/	0.10
	CTRL (n = 14)	95.0 (77.5-100.0)	100.0 (86.8-100.0)	100.0 (87.5-100.0)	100.0 (86.8-100.0)	3.226	0.358	/	0.08
General health	MI (n = 16)	55.0 (50.0-68.8)	67.5 (56.3-75.0)	65.0 (45.0-82.5)	60.0 (46.3-73.8)	4.787	0.188	/	0.10
	CTRL (n = 14)	72.5 (60.0-85.0)	77.5 (72.5-86.3)	82.5 (75.0-90.0)	77.5 (72.5-90.0)	15.110	0.002	T1 vs T3: p=0.0059	0.36

Data are presented as median (interquartile range). Within-group changes across time were assessed using Friedman's test, with post-hoc pairwise comparisons performed using Dunn's test where appropriate. Kendall's W is reported as a measure of effect size for repeated-measures analyses. Statistical significance was set at $p < 0.05$. .

Abbreviations: MI, myocardial infarction; CTRL, control group; CPET, cardiopulmonary exercise testing; VO_2peak, peak oxygen uptake; HR, heart rate; AT, anaerobic threshold (first ventilatory threshold); RCP, respiratory compensation point; VE/VCO_2 slope, ventilatory efficiency slope; VO_2/WR slope, oxygen uptake–work rate slope; VO_2/HR, oxygen pulse; LVEDd, left ventricular end-diastolic diameter; LVEF, left ventricular ejection fraction; IVS, interventricular septal thickness; PW, posterior wall thickness; E/A, ratio of early to late transmitral inflow velocities; E/e′, ratio of early transmitral inflow velocity to early diastolic mitral annular velocity; Triglycerides, serum triglycerides; HDL, high-density lipoprotein cholesterol; HDL/CHOL, high-density lipoprotein to total cholesterol ratio; LDL, low-density lipoprotein cholesterol; LDL/HDL, low-density lipoprotein to high-density lipoprotein ratio; fT3, free triiodothyronine; fT4, free thyroxine; NT-proBNP, N-terminal pro–B-type natriuretic peptide; CRP, C-reactive protein; hs-troponin, high-sensitivity cardiac troponin; eGFR, estimated glomerular filtration rate; TSH, thyroid-stimulating hormone; 6MWT, six-minute walking test; RL, role limitations.
